# Sex-dependent transcriptional and imaging responses to metformin in young-adult mice with SuHx-induced pulmonary arterial hypertension

**DOI:** 10.3389/fcvm.2026.1947678

**Published:** 2026-10-09

**Authors:** Irene Fernández-Folgueral, María Jesús Sánchez-Guisado, Irati Aiestaran-Zelaia, Marta Beraza, Aitor Zubillaga-Unsain, Claudia Miranda-Pérez de Alejo, Amanda Marrero-Pérez de Alejo, Kinsey Mae Thorpe, Angela Frimpong, Rui Adão, Ian J. Holt, Jesús Ruiz-Cabello

**Affiliations:** 1 Center for Cooperative Research in Biomaterials (CIC biomaGUNE), Basque Research and Technology Alliance (BRTA), Donostia-San Sebastián, Spain; 2 University of the Basque Country (EHU), Donostia-San Sebastian, Spain; 3 CIBERES, ISCIII (CIBER, Carlos III Institute, Spanish Ministry of Sciences and Innovation), Madrid, Spain; 4 Biogipuzkoa Health Research Institute, Donostia-San Sebastián, Spain; 5 Basque Centre on Cognition, Brain and Language (BCBL), Donostia-San Sebastián, Spain; 6 Department of Pharmacology and Toxicology, School of Medicine, Universidad Complutense de Madrid, Madrid, Spain; 7 Instituto de Investigación Sanitaria Gregorio Marañón, Madrid, Spain; 8 Ikerbasque, Basque Foundation for Science, Bilbo, Spain; 9 CIBERNED, ISCIII (CIBER, Carlos III Institute, Spanish Ministry of Sciences and Innovation), Madrid, Spain; 10 Department Chemistry in Pharmaceutical Sciences, Universidad Complutense Madrid, Madrid, Spain

**Keywords:** cardiac hypertrophy, glycolysis, metformin, pulmonary arterial hypertension, sex differences

## Abstract

Pulmonary arterial hypertension (PAH) is a progressive cardiopulmonary disease characterised by pulmonary vascular remodelling, right ventricular (RV) pressure overload, and ventricular metabolic reprogramming. Metformin has been proposed as a candidate therapy for PAH on the basis on encouraging preclinical results, most of which have been obtained in rat models and in single-sex cohorts. Its preventive effects in mice and its dependence on biological sex remain incompletely characterised. Based on the Sugen5416/hypoxia (SuHx) model, male and female mice were exposed to normoxia (Ctrl) or Sugen5416/Hypoxia (SuHx) conditions (PAH group) for 21 days, with two parallel groups, Ctrl + Met and PAH + Met, receiving metformin 0.25% w/v (effective dose 410–503 mg/kg/day) *ad libitum* in drinking water. The endpoints comprised cardiac cine and phase-contrast MRI, invasive RV systolic pressure (RVSP), Fulton index, [^18^F]-FDG PET, glucose and fatty acid related RT-qPCRs, histology and weekly measured body weight. These experimental data suggested that SuHx reliably induced RV mass gain and elevated RVSP in both sexes. Also, metformin did not attenuate the primary structural or haemodynamic features of the model in either sex. In fact, metformin triggers sex- and ventricle-specific responses detectable at multiple levels: a opposite transcriptional changes in RV's *Glut*4 between sexes, together with a coordinated downregulation of left ventricle (LV) fatty acid oxidation transcripts under both SuHx and metformin. Also, the histological analysis performed with WGA revealed that sex-specific cardiomyocyte hypertrophy phenotypes emerge only with metformin. Finally, the MRI determination of RV volumes and ejection fraction along with the pulmonary artery dilatation highlighted this sex-dysmorphism. Collectively, our data argue against a preventive benefit of metformin on the core SuHx-PAH phenotype in either male or female mice. Furthermore, they highlight cardiopulmonary and cellular markers that vary by sex and caution against the assumption that the beneficial effects of metformin in PAH models can be extrapolated without explicit confirmation stratified by sex.

## 1 Introduction

Pulmonary arterial hypertension (PAH) is a severe cardiopulmonary disorder characterised by pulmonary vascular remodelling, increased pulmonary vascular resistance, and progressive elevation of pulmonary arterial pressure (1, 2). The resultant increase in right-ventricular (RV) afterload induces compensatory hypertrophy and structural remodelling of the RV, which ultimately progresses to heart failure and represents the principal determinant of prognosis and survival in PAH (3). Despite recent advances in research, PAH remains a condition that reduces life expectancy and has a poor prognosis once RV dysfunction develops, highlighting the need for new therapies that address both pulmonary vascular disease and RV pathophysiology (1).

PAH is accompanied by profound metabolic reprogramming that affects not only the pulmonary vasculature but also the myocardium (3). In the RV, pressure overload promotes a metabolic shift from oxidative phosphorylation to the Warburg effect (4, 5). Such glucose utilization can be the result of impaired mitochondrial function; in any case, it reduces energetic efficiency and is associated with progressive contractile dysfunction (6, 7). Importantly, metabolic alterations are not always confined to the RV, as changes in glucose utilisation and systemic metabolism have also been observed in the left ventricle (LV) and peripheral tissues in PAH (3, 8, 9). Metabolomic characterization of PAH has identified alterations in glycolytic, glutamine, phospholipid, taurine, and fatty acid pathways at the levels of plasma, lung, and cardiac tissue in both patients (10–12) and preclinical rodent models (13–15).

Metformin (1,1-dimethylbiguanide hydrochloride), a first-line anti-diabetic agent, has attracted attention as a potential therapeutic candidate for PAH due to its pleiotropic metabolic effects (16, 17). Metformin primarily acts through inhibition of mitochondrial complex I of the respiratory chain, which raises the intracellular AMP:ATP ratio and thereby activates AMP-activated-protein kinase (AMPK), a master regulator of cellular energy homeostasis, that influences glucose metabolism, mitochondrial function, inflammatory signalling, and cellular proliferation (18, 19). In preclinical studies, metformin has demonstrated protective effects in several experimental models of pulmonary hypertension, including attenuation of pulmonary vascular remodelling, reduction of RV hypertrophy, and improvement of haemodynamic parameters (20–24). These findings have supported the growing interest in repurposing metformin as a metabolic-targeted therapy for PAH, an approach that has progressed to early phase-clinical evaluation with encouraging preliminary results (25).

However, significant uncertainties remain regarding the efficacy of metformin in PAH treatment. Many studies demonstrating the beneficial effects of metformin have been performed in rat models of pulmonary hypertension with metformin administered after disease establishment (20–24), whereas data from murine models, particularly under preventive treatment paradigms, are more variable (26, 27). Moreover, while biological sex has emerged as a critical modifier of PAH susceptibility, RV adaptation, and clinical outcome in humans, the so-called “sex paradox” of PAH (28–31), the sex-specific-effects of metformin remain incompletely characterised in preclinical PAH models (18).

The present study was designed to address these knowledge gaps by examining the preventive effects of metformin in a murine Sugen5416/hypoxia (SuHx) model of PAH, which reproduces key haemodynamic and structural features of the human disease, including RV pressure overload and pulmonary vascular remodelling (27, 32–34). Using an integrated *in vivo* phenotyping strategy combining longitudinal body-weight monitoring, positron emission tomography (PET), cardiac magnetic resonance imaging (MRI), invasive haemodynamic measurements, and pulmonary vascular histology, we aimed to determine whether preventive metformin treatment modulates the ventricular, haemodynamic, transcriptional and imaging responses to SuHx-induced PAH in mice of both sexes.

## 2 Materials and methods

### 2.1 Experimental mice model

All animal procedures were conducted in accordance with the European Union Directive 2010/63/EU and Spanish legislation (RD 53/2013) to protect animals used for scientific purposes. The experimental protocol was approved by the CIC biomaGUNE's Animal Ethical and Welfare Committee and the competent authority of the Basque Country (Diputación de Gipuzkoa; approval numbers PRO-AE-SS-259 and PRO-AE-SS-387).

A total of 82 C57BL/6J mice (42 females, 40 males; 8–10 weeks old mice, 20 g female–25 g male body weight average at study entry) were used for this study. Male and female mice of the PAH-representative groups were exposed to chronic normobaric hypoxia chamber (BioSpherix, Lacona, NY) (10% oxygen) for 3 weeks and were removed from the chamber only once a week for cleaning and the administration of intraperitoneal (IP) injection of the vascular endothelial growth factor (VEGF) inhibitor SU5416 or Sugen (MedKoo Biosciences, Inc.). Sugen was suspended in a vehicle consisting of 0.5% [w/v] carboxymethyl cellulose, 0.9% [w/v] sodium chloride, 0.4% [v/v] polysorbate 80, and 0.9% [v/v] benzyl alcohol in deionised water. Sugen was injected intraperitoneally weekly at a dose of 20 mg/kg body weight. Animals were allocated to four groups (Figure 1A), vehicle-treated normoxic controls (Ctrl), normoxic mice receiving metformin (Ctrl + Met), Sugen/hypoxia (SuHx) mice (PAH), and SuHx mice receiving metformin (PAH + Met), no group reported treatment-related mortality. The full cohort was distributed across complementary *in vivo* (MRI, PET, phase-contrast flow) and terminal *ex vivo* (invasive haemodynamic, Fulton index, pulmonary vascular morphometry, cardiomyocyte histology, RT-qPCR) techniques; not every animal underwent every procedure, so the *n* contributing to each analysis varies and is reported per group. *In vivo* endpoints (MRI/PET/flow) shared animals; terminal Ctrl/PAH endpoints (invasive haemodynamic, Fulton, vascular morphometry, RT-qPCR) came from separate cohorts; metformin animals span both; *n* per analysis varies depending on the technical difficulty or typical variability (Supplementary Table S1). In some cases, data were not used due to insufficient signal, blurring, presence of artefacts, or sample degradation.

**Figure 1:**
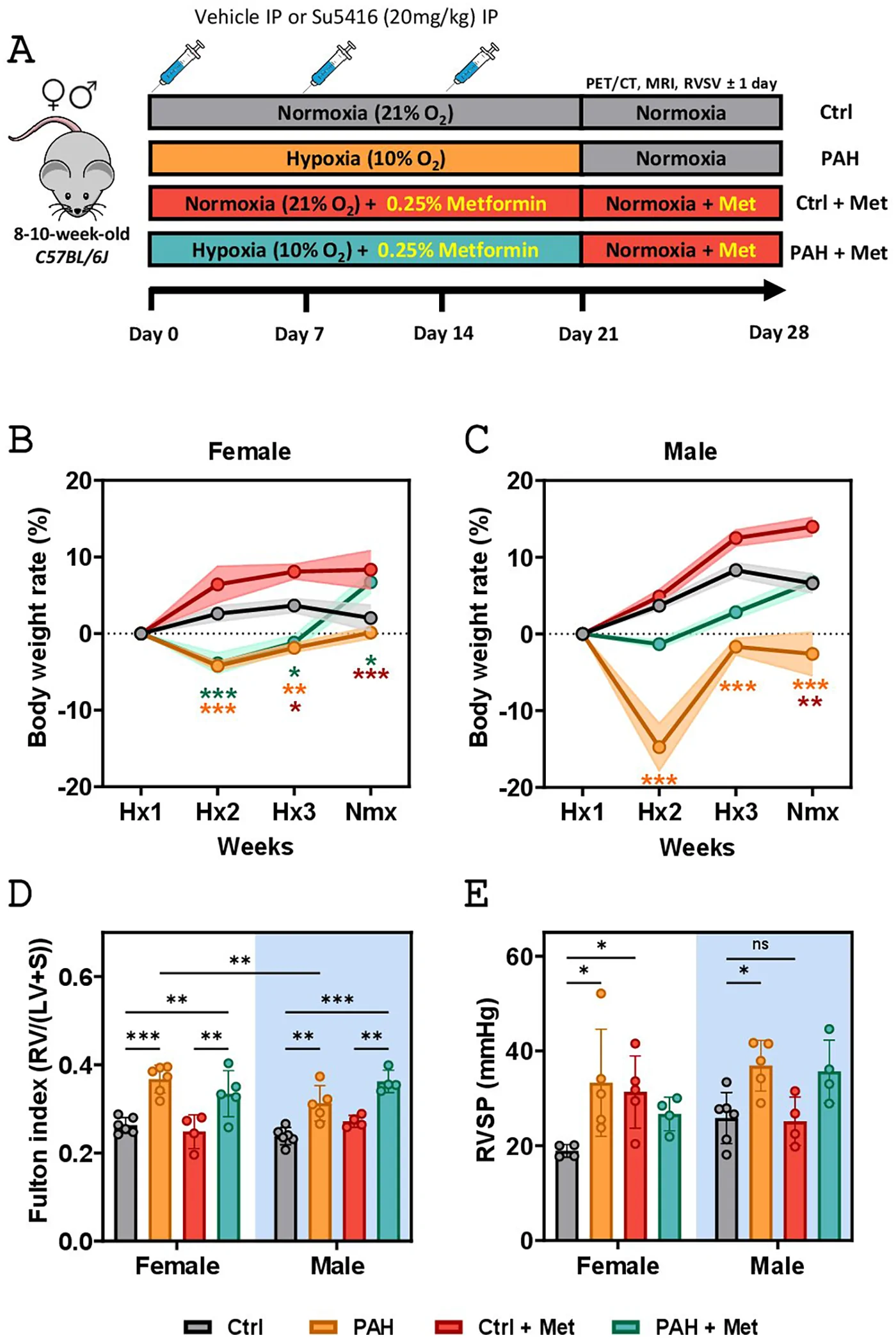
Metformin-treated animals present typical RV hypertrophy and pressure levels of PAH. **(A)** Representation of the experimental workflow used. Male and female mice were randomly assigned into the following groups: normoxia and weekly vehicle administration intraperitoneally (IP) (Ctrl); hypoxia with weekly Sugen IP (PAH); normoxia and metformin in the drinking water (Ctrl + Met); and hypoxia, Sugen IP and metformin in drinking water (PAH + Met). *In vivo* data collection: PET/CT, MRI and RVSP. Body weight change (%) over three weeks in **(B)** female and **(C)** male animals. Hx1, Hx2, Hx3: three weeks under hypoxic conditions. Nmx: normoxia week. N: Female: Ctrl = 12, PAH = 12, Ctrl + Met = 9, PAH + Met = 8; Male: Ctrl = 12, PAH = 12, Ctrl + Met = 8, PAH + Met = 8. Data are represented as mean ± SEM. **(D)** Fulton Index [Right ventricle (RV)/Left ventricle (LV) + septum] n: Female: Ctrl = 6, PAH = 6, Ctrl + Met = 4, PAH + Met = 5; Male: Ctrl = 6, PAH = 5, Ctrl + Met = 4, PAH + Met = 4. **(E)** RV systolic pressure (RVSP) n: Female: Ctrl = 4, PAH = 5, Ctrl + Met = 5, PAH + Met = 4; Male: Ctrl = 6, PAH = 5, Ctrl + Met = 4, PAH + Met = 4. Data are presented as mean ± SD. Statistical significance: *p < 0.05, **p < 0.01, ***p < 0.001, using GEE and two-way ANOVA *post-hoc* Šidák's correction (body weight) and Tukey's test (Fulton index and RVSP).

Metformin was administered *ad libitum* in drinking water at a concentration of 0.25% (w/v; 2.5 mg/mL) throughout the experimental period. Owing to these differences, the final effective doses were estimated based on the amount of drinking water consumed (Supplementary Table S2A). After three weeks of hypoxia exposure, all groups were characterised by [^18^F]-PET/Computer Tomography (CT) and MRI scans for the evaluation of glucose metabolism and haemodynamic measurements. Subsequently, tissues were collected for histological and molecular analyses.

### 2.2 Body weight, drinking, food consumption and metformin doses used in the study

Measurements were taken across four time points: three weeks under hypoxia (Hx1, Hx2, Hx3) and one week of recovery under normoxia (Nmx). Body weight (g) was recorded weekly for all animals throughout the experimental period, and the body weight change (BW%) was calculated as a percentage using the initial body weight (BW_0_) based on the following formula: BW% = ((BW_Week_ − BW_0_)/BW_0_) × 100. Water (Supplementary Table S2A) and food (Supplementary Table S2B) consumption were monitored weekly by weighing drinking bottles and food hoppers in each cage (Ctrl, PAH: *n* = 6 mice/cage, 1 cage/group; Ctrl + Met, PAH + Met: *n* = 3–6 mice/cage, 2 cages/group) and expressed as mean consumption per mouse per week (mean of 3–6 mice per group). One drinking measurement was excluded from the analysis (female Ctrl and PAH group, Hx2) because of a confirmed recording error in the laboratory notebook.

The effective metformin dose was estimated weekly for weeks 2–4 using the following formula: Dose (mg/kg/day) = [water intake (mL/mouse/week) × 2.5 (mg/mL)] ÷ [7 (days) × Body weight (kg)].

Water consumption differed according to sex and disease status (*r*). The effective dose was calculated individually based on measured daily water intake (2.8–4.9 mL/mouse/day) and individual body weight. The estimated effective metformin doses were 410 ± 22 mg/kg/day in male Ctrl + Met mice, 310 ± 13 mg/kg/day in male PAH + Met mice, 439 ± 39 mg/kg/day in female Ctrl + Met mice, and 503 ± 132 mg/kg/day in female PAH + Met mice. The estimated effective metformin dose (mg/kg/day) was calculated from the measured water intake and weekly body weight.

### 2.3 Radiochemistry, positron emission tomography scans and data acquisition

[^18^F]-2-fluoro-2-deoxy-D-glucose ([^18^F]-FDG) was provided by Curium Pharma (San Sebastian, Spain). PET and computed tomography (CT) scans were performed using Molecubes (Ghent, Belgium), *β*-CUBE (PET), and XCUBE (CT) systems. Radiotracer was administered intravenously. Before the injection, the animals were fasted for 12 h. Radioactivity (∼150 *μ*Ci) was injected, and after an uptake period of 30 min, the animals were re-anaesthetised and placed on PET for whole-body static acquisition in the 400–700 keV energetic window, with a total acquisition time of 10 min. Anaesthesia was induced by 4%–5% isoflurane inhalation in pure O_2_ and maintained with a 1.5%–2% flow rate based on respiration-rate monitoring (60–80 bpm). The animals were warmed using a pad installed inside the equipment. After each PET scan, a CT (140 mA intensity, 40 kV voltage) scan was performed to provide anatomical information for each animal and an attenuation map for subsequent PET image reconstruction. Reconstruction of the images was performed using the mathematical algorithm 3D-OSEM (3D ordered subset expectation maximisation; 30 iterations) and CT-based attenuation correction.

#### 2.3.1 PET image data analysis

PET/CT images were co-registered using PMOD image analysis software (PMOD Technologies Ltd., Zurich, Switzerland). Body weight, injected activity, injection time, correction factor, and scan time were used to convert the PET image units to standardised uptake values (SUV) according to standard methods. PET images were analysed using the open-source software ITK-SNAP (version 3.8.0). Regions of interest (ROIs) were manually drawn in the selected regions using the mouse CT image for each PET scan.

### 2.4 MRI acquisition

All MRI experiments were conducted using a 7 Tesla Bruker Biospec 70/30 USR MRI system (Bruker Biospin GmbH, Ettlingen, Germany) connected to an AVANCE III console and equipped with a BGA12-S imaging gradient insert (maximum gradient strength of 400 mT/m, switchable within 80 μs). Measurements were taken with a 72 mm volumetric quadrature coil for excitation and a 20 mm rat brain surface coil for reception.

Anaesthesia was induced with 3% isoflurane in 30% oxygen and maintained at 1%–2% isoflurane throughout the experiment. The animals were placed in a prone position on a customised 3D-printed bed with a head holder and kept warm with heated air circulated through an MRI-compatible system connected to a Monitoring and Gating Model 1025 (SA Instruments). The temperature (rectal) and respiration (via a respiratory pad) were monitored throughout the experiment.

#### 2.4.1 Cardiac magnetic resonance imaging

To ensure precise positioning, pure axial and 4-chamber view scout images were used to establish the representative heart view. For cardiac function measurements, short-axis images were acquired from the apex, covering both ventricles. A total of 11–12 slices, each 0.8 mm thick, were obtained using a Bruker cine self-gating so called IntraGate FLASH sequence with the following parameters: Effective echo time (TE) = 2.82 ms; repetition time (TR) = 6.87 ms; Matrix = 256 × 128 points; flip angle (FA) = 30°; field of view (FOV) = 25 × 25 mm. All images were reconstructed into 15 phases/frame of a single cardiac cycle. Cardiac MRI images were reconstructed into 15 cardiac phases using simultaneously acquired respiratory and cardiac motion signals, without the need for external gating hardware. For image analysis, short-axis cine MR images were divided according to the trigger time corresponding to each cardiac cycle period. ROIs were manually defined in these frames and slices using the open-source software ITK-SNAP (version 3.8.0). Endocardial and epicardial surfaces were manually segmented for both ventricles, including the papillary muscles and trabeculations, throughout the cardiac cycle. The images were segmented by two experienced researchers (IFF and JRC).

For each animal and chamber, the following scalars were computed from the realigned 100-point curve considering the heart rate (HR) and body surface area (BSA), which is indicated as an index (i):

- End-diastolic volume (EDV) = maximum curve (µL).
- End-systolic volume (ESV) = minimum curve (µL).
- Stroke volume (SV) = EDV − ESV (µL).
- Ejection fraction (EF) = 100 × SV/EDV (%).
- Cardiac output (CO) = (HR × SV)/1,000
- Cardiac index (CI) = (CO/BSA) × 10,000

#### 2.4.2 Phase-contrast magnetic resonance imaging

Phase-contrast MRI of the main pulmonary artery (MPA) was performed using a prospective double-gating sequence (Bruker BioSpin), with cardiac triggering synchronised to the R-wave of the electrocardiogram (ECG) and respiratory gating restricted to the end-expiratory phase, ensuring consistent velocity and flow measurements under stable haemodynamic and lung volume conditions. ECG triggering was achieved using subcutaneous needle electrodes (three-lead set, RMEC-703-4, SA Instruments, Inc., Stony Brook, NY, USA) inserted in the upper right and lower left limbs and connected to the same Monitoring and Gating Model 1025 (SA Instruments). Respiratory motion was recorded by placing a thoracic pressure pad over each animal. Imaging parameters were as follows: velocity encoding, 100 cm/s; FA 35°; slice thickness, 1.1 mm; bandwidth, 500.8 Hz; TE, 2.6 ms; TR, 5.5 ms; and 20 reconstructed cardiac phases. For accurate positioning, a four-chamber cine image was acquired to clearly visualise the MPA beneath the aortic arch. Based on this, a perpendicular imaging plane was selected to transect the MPA. This approach enabled reproducible placement of the imaging slice at the MPA trunk proximal to the first bifurcation.

#### 2.4.3 MPA flow quantification

The through-plane on the MPA cross-section was manually segmented on the magnitude image of each of the 20 cardiac frames using ITK-SNAP (v3.8.0). For every frame *k* (*k* = 1, …, 20), the mean signed velocity *v_k_* (cm·s^−1^) and cross-sectional area *A_k_* (cm^2^) within the ROIs were extracted. The per-frame instantaneous flow was *Q_k_* = *v_k_* · *A_k_* (mL·s^−1^). Forward and backward flow integrals were computed by trapezoidal integration with the frame index as the abscissa: FF = ∫ max(*Q_k_*, 0) d*k*; BF = ∫ max(−*Q_k_*, 0) d*k*.

SV per beat and CO were obtained from the recorded heart rate (HR, beats·min^−1^) as follows: Forward SV = FF · (60/(HR · 20)) mL; Net SV = (FF−BF) · (60/(HR · 20)) mL; and CO = Net SV · HR = (FF−BF) · 3 mL·min^−1^. Because exactly twenty frames cover one cardiac cycle by gated reconstruction, the per-frame duration scales as 1/HR, and CO expressed in this form is heart-rate-independent. Forward SV corresponds to the total volume ejected forward through the MPA per beat, the natural counterpart of the cine-derived RV SV in the absence of pulmonary regurgitation, while Net SV additionally subtracts retrograde flow. The backward fraction was expressed as BF (%) = BF/FF × 100. The median MPA cross-sectional area across the cycle was reported as a vessel calibre index. Velocity encoding was 100 cm·s^−1^; frames in which more than 1% of pixels in the ROIs lay within 1 cm·s^−1^ of ±velocity encoding (VENC) was flagged for aliasing review.

After quality control review, only three animals were excluded from group-level MPA-flow analyses, leaving 48 animals for flow and area measurements; final per-group *n* values are reported in the corresponding figure legends and tables.

### 2.5 Invasive hemodynamic measurements

At the end of the *in-vivo* imaging (MRI and [^18^F]-FDG PET/CT) performed during study days 24–28 (one-week normoxia), with individual acquisitions scheduled within a ±1 day window subject to scanner and radiotracer availability, all terminal endpoints, including hemodynamic and tissue collections were performed on the very last day of the experiment (Figure 1A). Owing to the intricate technical difficulties associated with directly measuring MPA pressure in mice, the right intraventricular pressure was used as a surrogate value. Mice were placed supine on a heated table under 2%–4% isoflurane inhaled anaesthesia. Using a closed-chest approach, the right neck area was shaved, and a small incision was made. The jugular vein was carefully dissected, and a tiny surgical opening was created for the insertion of a microcatheter (SPR-671NR Millar Catheter, ADInstruments, Dunedin, New Zealand). The catheter was then guided to the RV, which was confirmed by observing the characteristic square waveform, and measurements were promptly obtained. Right ventricular systolic pressure (RVSP) was recorded using a PowerLab data acquisition system (ADInstruments, Sydney, Australia) and digitised using the LabChart software (ADInstruments). The average RV pressure during systole was calculated to determine the individual RVSP.

### 2.6 Fulton index

After sacrifice, the hearts were excised and divided into RV and LV plus septum (LV + S). The RV to LV + S weight ratio and Fulton Index were computed to evaluate RV hypertrophy. Using this information, we calculated the Fulton index [RV mass/(LV + S mass)].

### 2.7 Histology

Tissue (heart and lung) samples were fixed with 10% formaldehyde for 48 h at room temperature with the lung inflated-fixed previously with formaldehyde through a syringe connected to the trachea and carefully inflated to total lung capacity. Subsequently, dehydration was performed using an alcoholic solution, followed by embedding in paraffin. 4 µm-thick sections were prepared and mounted on coverslips for histological assessments and comprehensive insights into tissue characteristics.

Lung sections were stained with Verhoeff-Van Giesson elastin stain to visualise elastic fibres in vessel walls. Tissue slides were maintained (15 min) in a working elastic stain solution (5% haematoxylin, 10% FeCl_2_, and Lugol's iodine). The slides were then washed with water after each of the following solutions: 2% FeCl_2%_ and 5% Na_2_S_2_O_3_. Next, they were submerged in the Van Gieson solution (5 min) and dehydrated in ethanol 95%. Pulmonary arteries (identified by accompanying airways) were imaged at 10× magnification using a Zeiss Axioscan 7 with ZEN slide scan 3.12 software.

About 16–20 pulmonary arteries were randomly selected for each lung section. These measurements enabled the calculation of the medial wall area (MWA) (%), following the formula: MWA (%) = (Total area − Lumen Area)/Total area × 100, as described in the literature (35). Additionally, the external diameter (ED) of each vessel was measured to characterise the arteries. Three MWA% strata were created based on the different ED observed: 1) >200 µm, 2) 200–100 µm; 3) 100–50 µm. All the measurements were analysed manually by QuPath software (version 0.7.0).

### 2.8 Immunofluorescence

For Wheat Germ Agglutinin (WGA) staining, paraffin-embedded heart sections (4 μm thick) were de-paraffiniased following standard procedures using xylene (5 min) and a graded ethanol series (3 min per step). Antigen retrieval was performed by immersing the samples in sodium citrate buffer (pH 6) at 95 °C for 30 min. Subsequently, the sections were incubated with FITC-conjugated lectin (Sigma, L-4895) diluted in PBS (100 μg/mL) for 90 min to visualise the cell membranes, as WGA specifically binds to membrane glycoproteins. Images were captured using an Axio Observer Z1 inverted fluorescence microscope (Zeiss).

Cardiomyocyte cross-sectional area was quantified from selected cells per animal in 3–4 animals per group. Per-animal mean values were calculated and used as the statistical units for between-group comparisons to avoid pseudoreplication from individual cell measurements. Nuclei within each ROI were stained with DAPI to aid visualisation and cell identification. Cardiomyocyte area was measured manually using ImageJ software (version 2.14), and cell nuclei were quantified using QuPath software (version 0.7.0).

### 2.9 Quantitative reverse transcription polymerase chain reaction (RT-qPCR)

The RV and LV for RNA analysis were excised and snap-frozen on dry ice and stored at −80°C. Total RNA was isolated from the tissue samples using the RNeasy Universal Plus Mini Kit (Qiagen) according to the manufacturer's instructions. RNA concentration and purity were assessed using a NanoDrop Ultra spectrophotometre (Thermo Fisher Scientific), and samples were stored at −80 °C until further use. cDNA was synthesised via reverse transcription from 600 ng of total RNA using the SuperScript VILO cDNA Synthesis Kit (Thermo Fisher Scientific) following the manufacturer's protocol. Reverse transcription conditions were 25 °C, 10 min; 42 °C, 60 min; 85 °C, 5 min; hold at 4 °C.

qPCR was performed using SYBR Green Master Mix (Applied Biosystems) on a Bio-Rad CFX96 Real-Time PCR System. cDNA was synthesised from 600 ng total RNA and diluted 1:10 prior to qPCR. Each reaction contained SYBR Green master mix, 1 µL of a premixed forward and reverse primer solution (Supplementary Table S3), 1 µL of cDNA template, and nuclease-free water to a final volume of 12.5 µL. Reactions were run under the following cycling conditions: 95 °C, 10 min; (95 °C, 15 s; 60 °C, 1 min) for 40 cycles.

### 2.10 Statistical analyses

Primary statistical analyses were performed using GraphPad Prism version 10 (GraphPad Software, San Diego, CA, USA). All analyses were independently reproduced in Python (v3.12; pandas, scipy, statsmodels, pingouin) and confirmed to match the GraphPad outputs. The analysis pipeline was reproducible end-to-end from the original data tables. Results are expressed as mean ± SD unless otherwise stated, and statistical significance was set at *α* = 0.05. Effect sizes for *post-hoc* comparisons were calculated in Python from the pooled error term of the corresponding two-way ANOVA and are reported as Cohen's *d* = (M_1_ − M_2_)/√MS_residual.

#### 2.10.1 Body weight and body weight change

Absolute body weight and BW% were analysed by generalised estimating equations (GEE; statsmodel v0.14, Python) with an exchangeable working correlation structure to account for repeated measurements within animals. Sex and Treatment were included as between-subject factors, time as a within-subject factor, and all two- and three-way interactions were tested. Inferences were based on Wald *χ*^2^ tests, and global effect sizes were reported as Wald *η*^2^ = *χ*^2^/(*χ*^2^ + df). Given the significant Sex × Treatment × Time interaction, planned pairwise comparisons were performed separately for each sex at each time point, using the pooled residual mean square from a two-way ANOVA (Sex × Treatment) refitted at the time point of interest and Šidák's correction across the twelve pairwise comparisons (six within-sex treatment contrasts × two sexes). The baseline time point was excluded from *post-hoc* testing because all BW% values were zero by definition.

#### 2.10.2 Food and fluid consumption

Weekly fluid and food consumption were recorded at the cage level and expressed as mL/mouse/week or g/mouse/week. Each group comprised one or two cages per time point, so the statistical unit was the cage rather than the individual animal; this precluded valid inferential testing, and consumption data are therefore presented descriptively as cage-level means per time point. Two values were excluded from the drinking-water dataset prior to analysis: the female Ctrl value at the first week of hypoxia was excluded after review of the original laboratory notebook confirmed a recording error, and the female PAH value at the third week of hypoxia was not available. These exclusions are stated in the relevant figure and table legends.

#### 2.10.3 Single-timepoint parameters

Parameters measured once per animal, PET-derived; cardiac MRI-derived; MPA velocity encoding indices; RVSP; Fulton index; and histological and RT-qPCR endpoints were analysed independently by two-way ANOVA (Sex × Treatment) with Type II sum of squares. *Post-hoc* pairwise comparisons were performed in GraphPad using Tukey's honest significant difference (HSD) test, with the pooled within-group mean square from the two-way ANOVA and the studentized-range distribution with *k* = 4 treatment groups for within-sex contrasts and *k* = 2 for cross-sex (female vs. male) contrasts within each treatment group. The effect sizes (Cohen's *d*) were calculated externally in Python using the same pooled MS_residual. For the RT-qPCR analysis, one value from PAH + Met females (*Glut*4) and one from PAH males (*Ldh*1) were identified as outliers by the GraphPad ROUT test (*Q* = 1%) and excluded from all analyses.

#### 2.10.4 Pulmonary vascular remodelling

To avoid pseudoreplication from multiple arteries per animal, statistical analyses were performed on the per-animal mean values. Each ED category was analysed independently by two-way ANOVA (sex × treatment), followed by Tukey's HSD *post-hoc* test.

### 2.11 Artificial intelligence use

Generative artificial intelligence to assist with writing and editing Copilot (Microsoft), was used to suggest linguistic improvements to the wording of the manuscript in English. Also, Claude (Opus 4.8) was also used to generate Python scripts to reproduce GraphPad-based described in methods 2.10 and 2.11, and to calculate effect sizes of all statistical analyses included in the paper. Also, Python scripts also generated representative images from all groups such as the systole and diastole behaviour of the heart (Figure 1) and MPA flow waveforms (Supplementary Figures S8–S10). All scripts are available under request to the authors.

## 3 Results

### 3.1 Sex-dependent effects of metformin on body weight evolution

Body weight trajectories differed substantially between sexes and groups across time (Figures 1B,C). In females (Figure 1B), the most striking pattern was the divergence between metformin-treated healthy animals and all other groups from Hx2 onwards. In females, Ctrl + Met progressively gained weight throughout hypoxia, whereas PAH and PAH + Met remained near or below their baseline weight. This divergence was large in magnitude by Hx3; Ctrl + Met females had gained approximately 8% above baseline while PAH females had lost approximately 2% (*d* = 1.87, vs. Ctrl), and PAH + Met presented a similar profile to the latter (*d* = 1.64). Importantly, PAH + Met did not differ from untreated PAH at Hx2 or Hx3, indicating that metformin did not attenuate body weight decline associated with SuHx-induced disease in females. With Nmx, PAH + Met showed partial recovery toward Ctrl levels, whereas PAH females remained significantly below Ctrl + Met animals (*d* = 1.45), suggesting incomplete spontaneous weight restoration in the untreated disease group.

In males, the pattern was notably different and sexually dimorphic (Figure 1C). The most dramatic finding was the acute and severe body weight loss in PAH males at Hx2 (−14.7%), a magnitude approximately four times greater than the modest gain observed in Ctrl males at the same time point (*d* = 3.52), and consistent with the known acute systemic response to SuHx. Despite this marked initial loss, males with PAH showed subsequent recovery with no clinical signs of deterioration throughout the experimental period. The key therapeutic observation was the near-complete attenuation of this acute weight loss in PAH + Met at Hx2 (−1.3%), whose BW% remained close to baseline and differed from untreated PAH males by over 15% (*d* = −2.5). By Hx3, PAH males had largely recovered toward baseline, narrowing the gap with PAH + Met males, and the two disease groups were no longer distinguishable from one another. The dominant pattern at Hx3 was continued weight gain in Ctrl + Met males (+12.5%), which diverged from both PAH (*d* = 2.9) and PAH + Met males (*d* = 1.27).

At normoxia, this divergence between Ctrl + Met males and the disease groups persisted, whereas PAH and PAH + Met males remained indistinguishable, suggesting that metformin's early protective effect on body weight was not sustained as an independent advantage under normoxic recovery conditions. Taken together, the male data revealed that metformin's most striking impact on body weight was its attenuation of acute SuHx-induced weight loss at the onset of hypoxia, an effect that was both large in magnitude and entirely absent in females, highlighting a clear sexual dysmorphic response to metformin in this model.

### 3.2 Metformin-treated mice exhibit key signs of RV hypertrophy characteristic of PAH

RV hypertrophy was assessed by the Fulton index (Figure 1D, Table 1). Two-way ANOVA revealed a large effect of Treatment (*η*^2^*p* = 0.71), principally driven by the SuHx-induced increase in RV mass and a significant Sex × Treatment interaction indicating sex-dependent modulation by metformin (*η*^2^*p* = 0.26, Table 1), indicating that the pattern across the four groups differed between sexes. This interaction should not be interpreted as evidence of a beneficial modulation by metformin. As detailed below, in neither sex did metformin significantly attenuate the Fulton index in PAH animals, and in males PAH + Met values were numerically higher than in PAH alone.

**Table 1:** Two-way ANOVA of *in vivo* and end-point parameters.

	Two-way ANOVA					
	Sex		Treatment		Sex × treatment	
Parameter	*p*-value	*η* ^2^ *p*	*p*-value	*η* ^2^ *p*	*p*-value	*η* ^2^ *p*
Fulton index [RV/(LV + S)]	0.262 ns	0.039	<0.001***	0.713	0.02*	0.262
RVSP (mmHg)	0.146 ns	0.072	0.002**	0.386	0.10 ns	0.191
RV [^18^F]-FDG SUVmean	0.697 ns	0.005	<0.001***	0.553	0.075 ns	0.181
LV [^18^F]-FDG SUVmean	0.19 ns	0.066	0.004**	0.323	0.082 ns	0.177

Fulton Index [Right ventricle (RV)/Left ventricle (LV) + septum] n: Female: Ctrl = 6, PAH = 6, Ctrl + Met = 4, PAH + Met = 5; Male: Ctrl = 6, PAH = 5, Ctrl + Met = 4, PAH + Met = 4. RV systolic pressure (RVSP) n: Female: Ctrl = 4, PAH = 5, Ctrl + Met = 5, PAH + Met = 4; Male: Ctrl = 6, PAH = 5, Ctrl + Met = 4, PAH + Met = 4. Statistical significance: *p < 0.05, **p < 0.01, ***p < 0.001, ns, not significant; assessed using two-way ANOVA *post-hoc* Tukey test. One animal excluded after curve-quality review.

In females, both PAH and PAH + Met animals showed substantially elevated Fulton indices relative to Ctrl (*d* = −3.29 and *d* = −2.25, respectively) consistent with robust indication of RV hypertrophy by SuHx. Therefore, metformin did not attenuate RV hypertrophy response since PAH + Met and PAH animals were indistinguishable (*d* =  +1.04).

In males, the pattern differed. The PAH + Met group displayed the highest Fulton index of the cohort, substantially exceeding Ctrl (*d* = −3.9) and Ctrl + Met animals (*d* = −2.84), and numerically, though not significantly, higher than untreated PAH males (0.31 ± 0.04, *d* = −1.56) (Figure 1D). Rather than the expected attenuation of hypertrophy, PAH + Met males therefore showed a paradoxical numerical elevation relative to PAH, an observation we interpret cautiously given that the direct PAH vs. PAH + Met contrast did not reach statistically significance, but that nonetheless argues against a beneficial cardio-protective action of metformin on RV hypertrophy in the male SuHx heart.

Given that RV hypertrophy is downstream of pulmonary pressure elevation, RVSP was measured to determine whether these structural observations were accompanied by corresponding haemodynamic changes (Figure 1C). The two-way ANOVA showed a significant effect of Treatment (*η*^2^*p* = 0.39), principally reflecting SuHx-induced RVSP elevation; no significant effect of Sex (*η*^2^*p* = 0.07) and no significant Sex × Treatment interaction (*η*^2^*p* = 0.19) (Table 1). Consistent with successful induction of the PAH phenotype, both female and male PAH animals showed elevated RVSP relative to Ctrl (females: *d* = −2.18; males: *d* = −1.67), although neither pairwise contrast reached Tukey-corrected significance within its own sex. Metformin did not attenuate RVSP in PAH animals of either sex (females: PAH vs. PAH + Met: *d* =  + 0.79; males: *d* =  + 0.20; both).

Two additional observations in the Ctrl + Met group deserve mention. In male animals, metformin alone was associated with a small elevation of the Fulton index relative to Ctrl (*d* = −2.03), that did not reach significance. In female animals, Ctrl + Met was associated with a numerically elevated RVSP comparable to that of PAH females and substantially above female Ctrl (31.3 vs. 18.9 mmHg, *d* = −1.89), an effect not observed in males (*d* =  + 0.10) and again not surviving multiple-testing correction. Although these Ctrl + Met observations are secondary and not the focus of the study, they suggest that metformin administration in healthy animals may itself elicit small ventricular and haemodynamic responses in a sex-dependent manner and warrant explicit acknowledgement.

Taken together, the Fulton index and RVSP data indicate that the mean structural and haemodynamic features of experimental PAH were not attenuated by metformin in either sex, or that in male animals PAH + Met showed evidence of paradoxically worsened RV remodelling.

### 3.3 Differential cardiomyocyte remodelling in response to metformin

The increase in Fulton index observed in the SuHx groups indicates RV mass gain in both sexes, and we assessed whether this was accompanied by cardiomyocyte hypertrophy at the histological level (Supplementary Figure S2). In males, RV cardiomyocyte cross-sectional area tended to be higher in the PAH and PAH + Met groups than in Control, suggesting a hypertrophic contribution to the observed RV mass gain, although none of the between-treatment WGA comparisons reached statistical significance (Supplementary Figure S2B). LV cardiomyocyte area in males was essentially unchanged across all four groups, consistent with the RV-specific loading imposed by SuHx (Supplementary Figure S2C).

In females, RV cardiomyocyte area (Supplementary Figure S2B) remained stable in PAH vs. Control despite a marked increase in Fulton index (Figure 1), pointing to a dissociation between RV mass gain and cardiomyocyte size in this sex. A distinct pattern was observed in the female LV (Supplementary Figure S2C), where cardiomyocyte area was significantly smaller in the metformin-treated groups than in the corresponding non-treated groups (Ctrl vs. Ctrl + Met: *d* = 2.94; PAH vs. PAH + Met: *d* = 1.64) and was accompanied by a corresponding increase in cardiomyocyte density (Ctrl vs. Ctrl + Met: *d* = −1.14).

A sex-specific effect was particularly evident in the metformin-treated groups. In the PAH + Met condition, male cardiomyocyte area was 55% larger than their female counterparts in the RV (*d* = 3.21) and 51% larger in the LV (*d* = 3.69) (Supplementary Figures S2B,C). These sex differences were absent in the corresponding non-treated groups, where male and female cardiomyocyte areas were comparable. Consistent with these area differences, RV and LV cardiomyocyte density in metformin-treated groups tended to be higher in females than in males (Supplementary Figures S2D,E), likely reflecting the geometric consequence of smaller cell size in the female metformin groups.

Taken together, these observations suggest that under metformin treatment male and female hearts converge to markedly different cardiomyocyte size phenotypes in both ventricles, an effect that is not detected in animals without the drug. Despite that is not possible to attribute the RV mass changes to a single cellular mechanism common to both sexes and ventricles, the RV mass gain in females is not accompanied by a proportional increase in cardiomyocyte cross-sectional area. This raises the possibility that mechanisms, other than pure cardiomyocyte hypertrophy (including changes in cell number, extracellular matrix expansion, or interstitial fluid) contribute to it.

### 3.4 Experimental PAH drives sex-specific myocardial glucose uptake reprogramming that is insensitive to metformin

[^18^F]-FDG PET/CT was used to assess the myocardial glucose uptake response to SuHx-induced PAH and its potential modulation by metformin. The overall pattern was consistent across both ventricles: glucose uptake was elevated in the disease groups and was unaffected by metformin treatment.

In the RV (Figures 2A,B), glucose uptake rose markedly with disease in females, RV SUV_mean_ increased from 1.4 in Ctrl to 3.0 in PAH (*d* = −2.7). In males the corresponding change was smaller (1.5 to 2.1), despite a noticeable increase in glucose uptake (*d* = −1.13). [^18^F]-FDG uptake remained elevated in PAH + Met animals of both sexes relative to their respective controls. Moreover, these PAH metformin-treated groups showed elevated uptake relative to Ctrl + Met in both sexes (female: *d* = −2.06; male: *d* = −1.9). Meanwhile PAH and PAH + Met did not differ, indicating the lack of glucose influx mitigation. Although only PAH + Met males reached significance regarding the control (*d* = −2.28), the persistence of high glucose uptake in the metformin-treated disease animals of both sexes confirms that the treatment did not reverse the disease-associated increase in RV glucose uptake.

**Figure 2:**
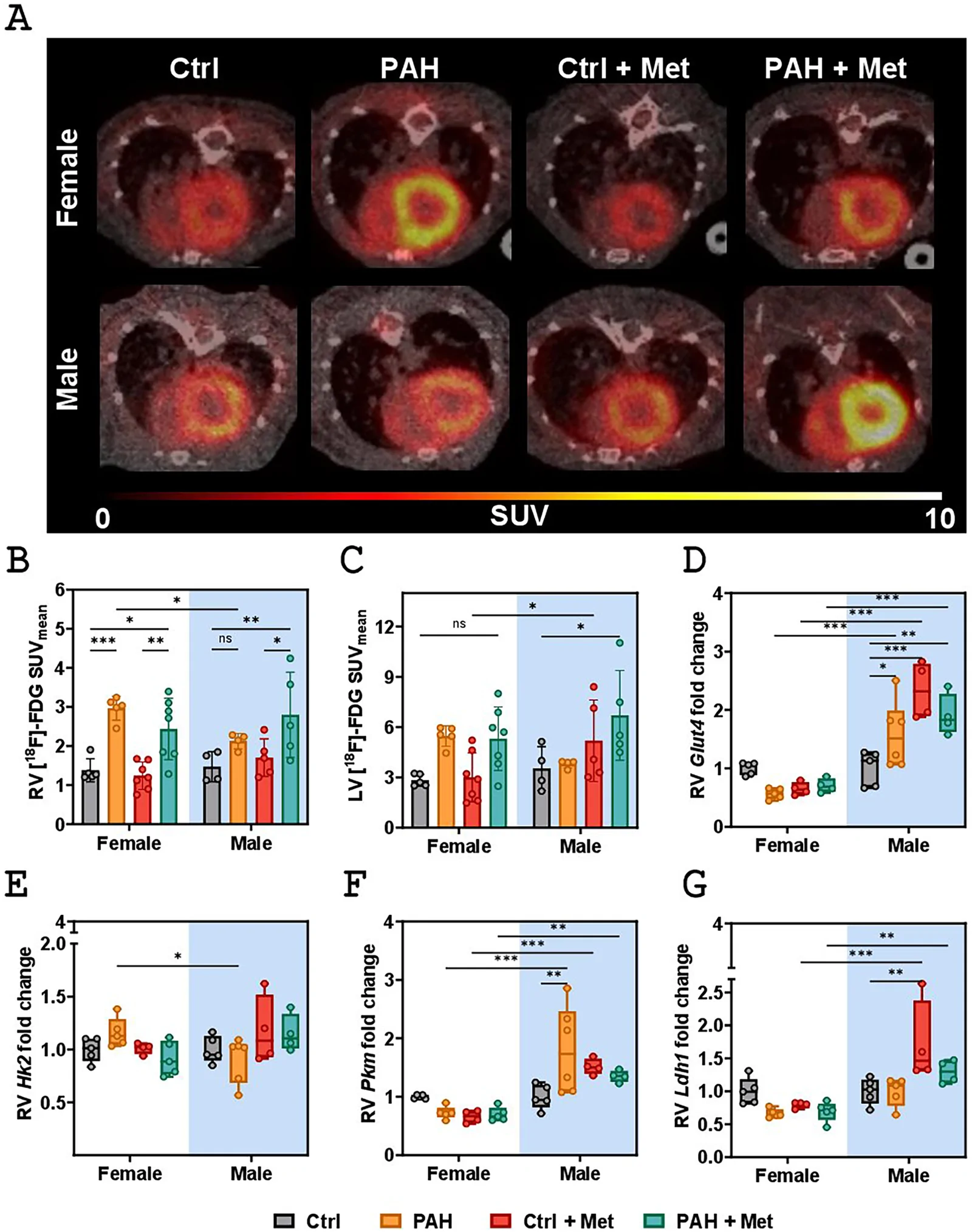
PAH + Met exhibits similar glucose utilization as the non-treated PAH group. **(A)** Representative axial positron emission tomography (PET) images of the middle heart fused with corresponding computed tomography (CT), data of mice administered [^18^F]-FDG. Measurement of SUV uptake in the **(B)** right ventricle (RV) and **(C)** left ventricle (LV). n: Female: Ctrl = 5, PAH = 5, Ctrl + Met = 7, PAH + Met = 7; Male: Ctrl = 4, PAH = 4, Ctrl + Met = 5, PAH + Met = 5. Relative fold change in mRNA from RV obtained through RT-qPCR of **(D)** *slc*2*a*4 (*Glut*4) one female PAH + Met sample was excluded, **(E)** *hk*2, **(G)** *pkm* and **(G)** *ldh-*1 where one male PAH sample was excluded. Values are presented as mean ± SD. N: Female: Ctrl = 5, PAH = 5, Ctrl + Met = 4, PAH + Met = 4; Male: Ctrl = 5, PAH = 4, Ctrl + Met = 4, PAH + Met = 4. Statistical significance: *p < 0.05, **p < 0.01, ***p < 0.001, according to the evaluation performed using two-way ANOVA *post-hoc* Tukey's test. Values excluded were positive at ROUT test.

A comparable but weaker pattern was observed in the LV (Figure 2C). LV SUV_mean_ trended upward in female PAH (*d* = −1.58) and PAH + Met (*d* = −1.48) animals without reaching significance regarding the control. In males, only the PAH + Met group was significantly elevated (6.7 vs. 3.5 in Ctrl). The highest LV uptake was in the metformin-treated disease groups, again indicating that the drug did not normalise myocardial glucose incorporation.

To investigate glucose utilization in the RV in greater detail, we profiled four genes governing glucose handling: solute carrier family 2 member 4 (*Glut*4), the first glycolytic enzyme hexokinase 2 (*Hk*2), pyruvate kinase (*Pkm*), and lactate dehydrogenase 1 (*Ldh*1), which diverts pyruvate away from the mitochondria by converting it to lactate (Figures 2D–G). It was anticipated that PAH would enhance the glycolytic transcriptional program, consistent with the elevated aerobic glycolysis reported in experimental PAH models (6, 7). However, the glucose-related transcriptional responses did not uniformly follow this prediction and diverged markedly between the sexes. In females, *Glut*4, *Pkm* and *Ldh*1 were all repressed in the PAH groups (*Glut*4 and *Ldh*1: *d* = 1.3; *Pkm*: *d* = 0.08) (Figures 2D–G), with a similar suppression observed in females treated with metformin alone (*Glut*4 and *Pkm*: *d* = 1; *Ldh*1: *d* = 0.82). This pattern suggests that these three markers are sensitive to altered glucose homeostasis in female murine hearts. Importantly, their downregulation should not be interpreted as indicative of reduced glycolysis, given that glucose uptake, as measured by PET, doubled in PAH females.

In males, the same three genes responded in the opposite direction, with *Glut*4, *Pkm*, and *Ldh*1 all increasing in expression with experimental PAH (*Glut*4: *d* = −1.75; *Pkm*: *d* = −2.48; *Ldh*1: *d* = 0.03) (Figures 2D–G). As with the PET data, the gene expression profiles thus diverged between the sexes in response to PAH. Unexpectedly, metformin produced similar changes in gene expression as PAH for *Glut*4, *Pkm*, and *Ldh*1, here again in opposite directions in the two sexes (Figures 2D–G). Hence, it is no surprise that metformin did not normalise myocardial glucose utilisation.

The fourth enzyme tested, *Hk*2, was an outlier in several ways; rather than decreasing in the female RV, its expression marginally increased, and uniquely, its expression was restored to control levels by metformin in experimental PAH (*d* = −0.9) (Figure 2E). While *Hk*2 responded differently to PAH between males and females, as per the other three enzymes, the direction was the opposite to the others, as it increased in females and decreased in males (Figure 2E). However, the changes in all conditions were small; therefore, *Hk*2 does not appear to be a useful marker of altered glucose homeostasis in experimental PAH.

Together, these findings reveal a clear dissociation between myocardial glucose uptake and glycolytic gene expression in the female RV: [^18^F]-FDG uptake increased with disease, whereas the transcripts *of Glut*4 and glycolytic enzymes decreased. At the level of glucose uptake, however, the failure of metformin to normalise the PAH-associated [^18^F]-FDG signal was evident in both sexes, indicating that the drug does not reverse this imaging-based phenotype of the model, and that this axis does not, by itself, account for the marked sex-divergence of metformin's effects on body weight and RV structure described.

To extend the transcriptional characterisation of ventricular substrate handling, we expanded the panel to include four genes involved in mitochondrial fatty acid uptake and *β*-oxidation. We selected two transporters, *Cpt*1*a* (carnitine palmitoyl transferase 1a, outer mitochondrial membrane) and *Ucp*3 (uncoupling protein 3, inner mitochondrial membrane), and two acyl-CoA dehydrogenases that catalyse the first step of *β*-oxidation for very-long-chain (*Acadvl*) and short-chain (*Acads*) fatty acids.

In the RV, fatty acid oxidation transcripts remained largely unchanged across all groups and in both sexes; only a single contrast reached significance when comparing the two treated groups (male *Acadvl* Ctrl + Met vs. PAH + Met, *d* = 2.17), consistent with the previously reported concentration of RV transcriptional changes on the glycolytic side (Supplementary Figures S11A,C,E,G). In sharp contrast, the LV showed a robust and largely uniform downregulation of fatty acid oxidation transcripts across metformin- and SuHx-exposed groups (Supplementary Figures S11B,D,F,H). *Cpt*1*a* was reduced in Ctrl + Met vs. Control in both sexes (females *d* = 2.58; males *d* = 2.15), with PAH + Met values recovering towards baseline in females and partially in males. *Ucp*3 showed the strongest and most consistent effect: expression was significantly reduced (60%–83%) all groups vs. Control in both sexes. Among the acyl-CoA dehydrogenases, *Acadvl* showed a modest 22%–25% reduction in female and male PAH + Met LV, and *Acads* was significantly reduced in the LV of both sexes in the PAH + Met group vs. Control (female: *d* = 1.94; male *d*: = 1.92), with an additional PAH vs. PAH + Met reduction in males (*d* = 1.75).

Whereas the glycolysis-related changes reported above were concentrated in the RV, notably the *Glut*4 collapse in the young female RV, the fatty acid oxidation transcriptional signature was localised to the LV and pointed uniformly in the direction of reduced substrate uptake and *β*-oxidation gene expression under both metformin and SuHx exposure. Together, the two datasets suggest a coordinated, but ventricle compartmentalised transcriptional signature of substrate handling in the pressure-overloaded and metformin-treated heart, with the RV and LV contributing complementary evidence at the level of transcript abundance.

### 3.5 Metformin does not confer cardiac benefit in the female RV

To characterise how the established RV hypertrophy translated into ventricular geometry and pump function, and whether metformin altered the cardiac phenotype, we performed cine cardiac MRI. (Figure 3) As expected, males had larger LV and RV volumes than females in most groups (Figure 4, Table 2, Figure 3, and Supplementary Figure S3). The Sex × Treatment interaction did not formally show clear significance, but the pattern was consistent across LV EDV, LV ESV, RV EDV, and RV ESV (Table 2 and Supplementary Figure S4), with sex differentiation across groups, which was reflected by RV EDV (*η*^2^*p* = 0.27).

**Figure 3:**
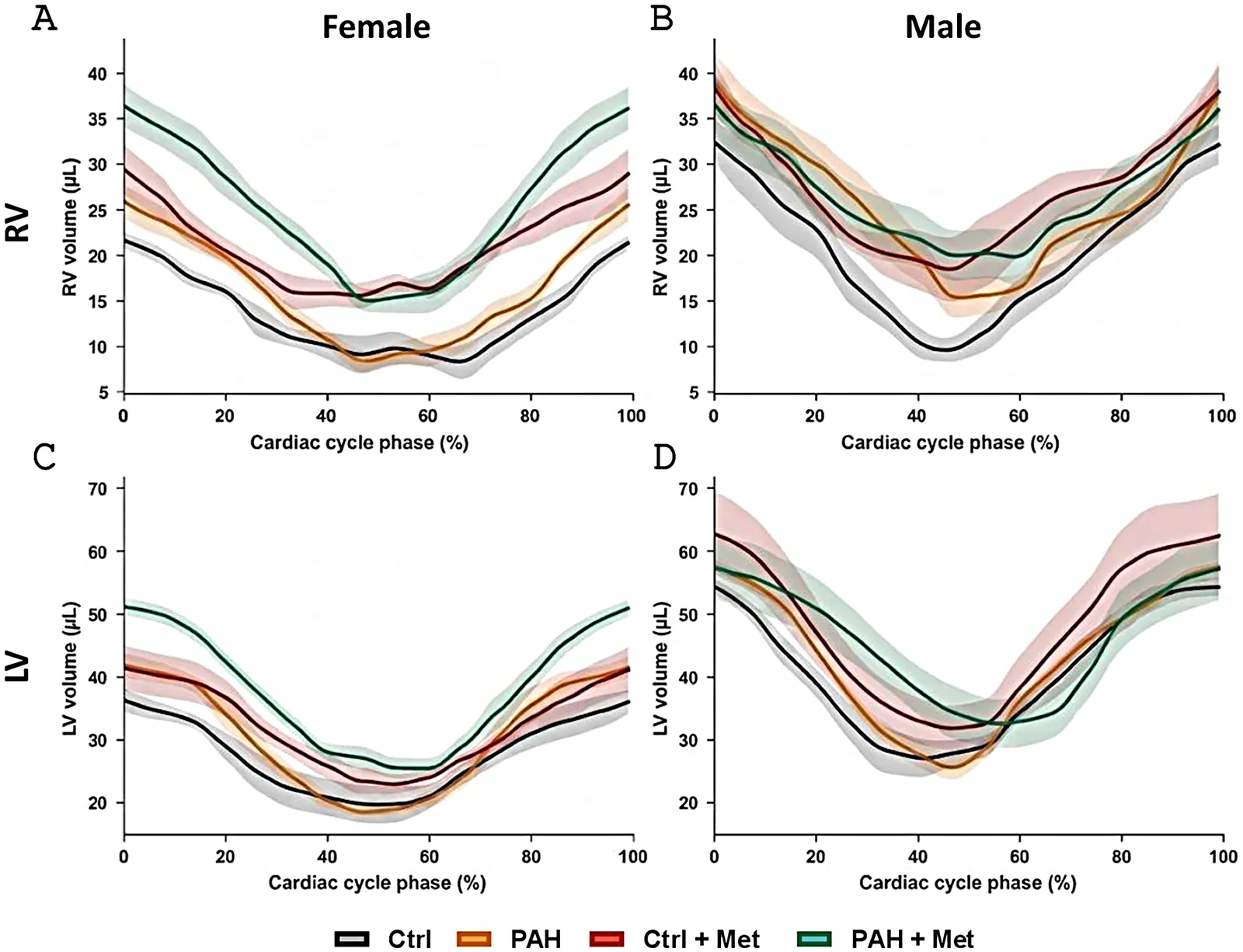
Phase-aligned cine MRI volume curves across the cardiac cycle. Volume-time curves (Mean ± SEM) over one cardiac cycle for the **(A,B)** right ventricle (RV) and **(C,D)** left ventricle (LV) in **(A,C)** female and **(B,D)** male mice. Curves were phase-aligned per animal so that end-diastole (ED) sits at phase 0, then resampled to 100 equally spaced phase points. Shaded ribbons denote ± 1 SEM around the group mean. N: Female: Ctrl = 5, PAH = 5, Ctrl + Met = 7, PAH + Met = 9; Male: Ctrl = 5, PAH = 5, Ctrl + Met = 6, PAH + Met = 5. Statistical comparisons of derived scalars are reported in Figure 4.

**Figure 4:**
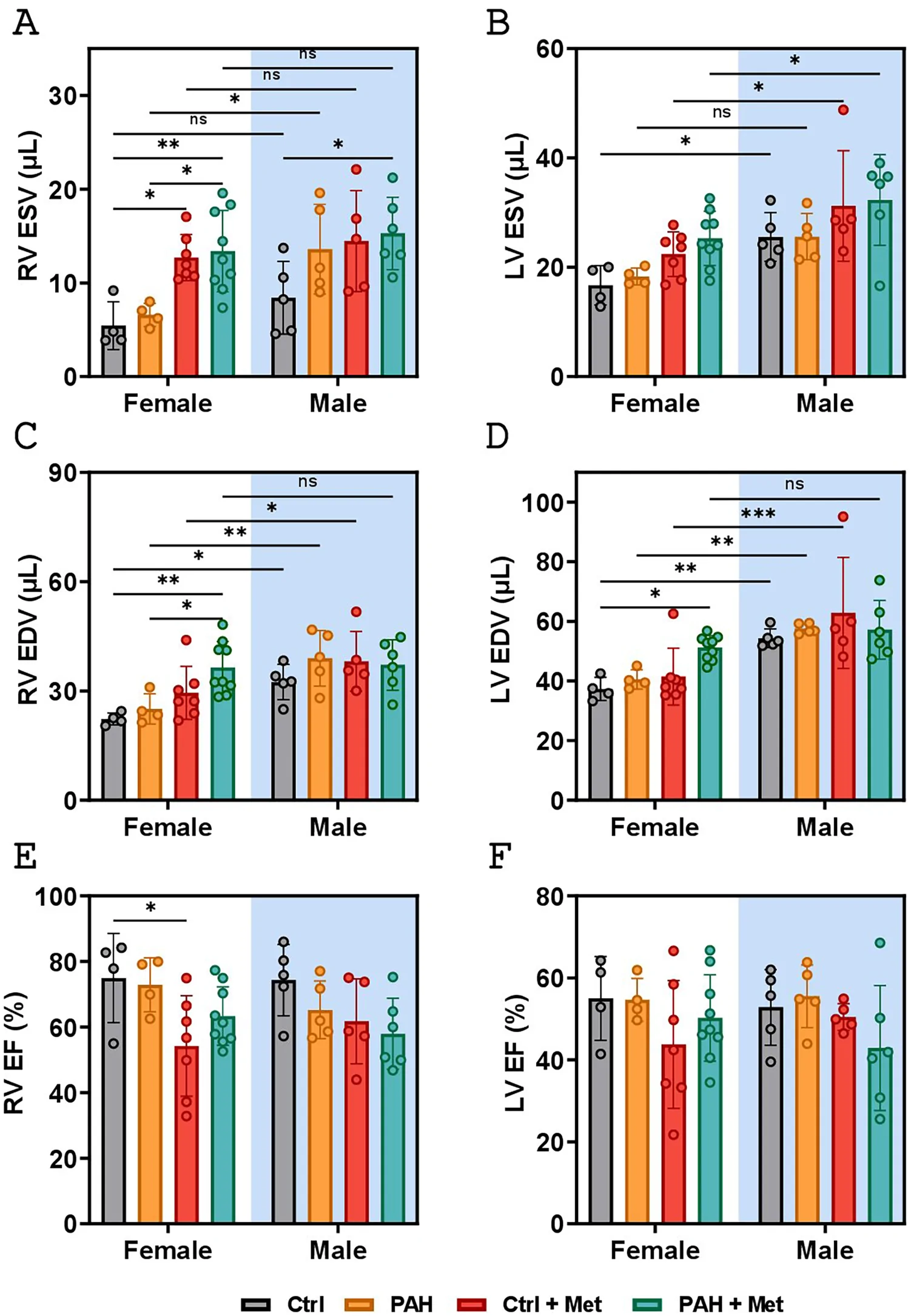
Male show higher volumetric responses than female. **(A,C,E)** Right ventricle (RV) and **(B,D,F)** left ventricle (LV): **(A,B)** End-Systolic Volume (ESV); **(C,D)** End-Diastolic Volume (EDV); **(E,F)** Ejection Fraction percentage (EF%). n: Female: Ctrl = 4, Ctrl + Met = 7, PAH = 4, PAH + Met = 9; Male: Ctrl = 5, Ctrl + Met = 5, PAH = 5, PAH + Met = 5. Data are presented as the mean ± SD. Statistical significance: *p < 0.05, **p < 0.01, ***p < 0.001, assessed using two-way ANOVA *post-hoc* Tukey test.

**Table 2:** Two-way ANOVA of curve-derived functional cardiac MRI parameters.

	Two-way ANOVA					
	Sex		Treatment		Sex × treatment	
Parameter	*p*-value	*η* ^2^ *p*	*p*-value	*η* ^2^ *p*	*p*-value	*η* ^2^ *p*
HR (bpm)	0.052 ns	0.136	0.013*	0.218	0.740 ns	0.020
RV EDV (µL)	<0.001***	0.270	0.004**	0.257	0.100 ns	0.145
RV ESV (µL)	0.016*	0.162	<0.001***	0.405	0.200 ns	0.074
RV EF (%)	0.914 ns	0.005	0.037*	0.279	0.340 ns	0.073
RV SV (µL)	0.028*	0.1233	0.611 ns	0.047	0.263 ns	0.1
RV CO (mL/min)	0.01*	0.16	0.64 ns	0.04	0.27 ns	0.098
RV SV/ESV (coupling)	0.647 ns	0.003	0.007**	0.273	0.368 ns	0.040
LV EDV (µL)	<0.001***	0.465	0.043*	0.158	0.145 ns	0.137
LV ESV (µL)	<0.001***	0.356	0.016*	0.273	0.902 ns	0.007
LV EF (%)	0.698 ns	0.002	0.332 ns	0.121	0.634 ns	0.068
LV SV (µL)	0.008**	0.176	0.88 ns	0.018	0.155 ns	0.13
LV CO (mL/min)	0.001**	0.243	0.93 ns	0.012	0.12 ns	0.142

HR, heart rate; Trt, treatment; LV, left ventricle; RV, right ventricle; EDV, end-diastolic volume; ESV, end-systolic volume (µL); EF, ejection fraction (%); SV, stroke volume. Values are derived from phase-aligned, 100-point cine volume–time curves. n: Female: Ctrl = 4, PAH = 4, Ctrl + Met = 7, PAH + Met = 9; Male: Ctrl = 5, PAH = 5, Ctrl + Met = 5, PAH + Met = 6. Statistical significance: *p < 0.05, **p < 0.01, ***p < 0.001, ns, not significant; assessed using two-way ANOVA *post-hoc* Tukey test.

The male-female gap in absolute volumes was present in every treatment group, except PAH + Met, across the various parameters analysed (Figure 4 and Supplementary Figure S4). In PAH + Met, the RV EDV in females and males was comparable (36.4 vs. 37.1 µL), the difference in LV EDV was halved, and the sex differences in LV ESV and RV EDV were no longer statistically significant (Figure 4 and Supplementary Figure S4). When ventricular volumes were normalised to BSA (Supplementary Figure S5), the male-female difference persisted on the LV EDVi but dropped to borderline significance for the RV EDVi and ESVi. COi was sex-equivalent in both ventricles, except for a decrease in Ctrl + Met females compared to their male counterparts (*d* = −0.78). The LV is therefore unequivocally sex-dimorphic, whereas RV size is largely a function of body size.

RV showed the most consistent response to metformin. In females, the RV ESV doubled from Ctrl to PAH + Met (*d* = −2.05) and from PAH to PAH + Met (*d* = −1.75) (Figure 4). Strikingly, female metformin alone produced changes comparable to the PAH + Met group: RV ESV from Ctrl + Met was twice that of Ctrl (*d* = −1.87) and significantly higher than that of PAH (*d* = 1.58). In males, RV ESV rose with metformin in normoxic animals, similar to the increase in PAH + Met. The RV EDV showed the same trend without reaching significance. In females, the PAH + Met presented higher amounts of blood remaining inside the RV after systole and diastole compared to PAH alone, which is indicative of metformin further reducing blood flow in experimental PAH.

Focusing on LV EDV in females, it rose by 37% in PAH + Met (*d* = −1.63) compared to the control, indicating increased preload. Ctrl + Met (41.5 µL) and PAH (40.5 µL) sat between, suggesting both metformin and the model contributed to this increase. In males, LV EDV did not differ across treatments. The LV ESV moved in the same direction without reaching statistical significance, indicating a similar pattern. LV ESV was elevated in the metformin-receiving groups, but the *post-hoc* contrasts did not show a clear correction (Figure 4 and Supplementary Figure S4). Attending the RV EF%, it dropped by 20% in PAH metformin-treated females. Meanwhile, male RV EF% showed a parallel drop (Ctrl 74.4% to PAH + Met 61.7%) that did not reach significance. The LV EF% was not adversely affected by PAH across both sexes. Given that there were no significant differences in RV EF% between the sexes, it was most appropriate to interpret these data as a single metformin-related reduction in RV systolic function common to both sexes.

From the analysis of ventricular volume curves (Figure 3) obtained from cardiac MRI images, the SV and CO were calculated and are summarised in Table 2. These parameters exhibited significant sex differences in the two-way ANOVA (SV: *η*^2^*p* = 0.19, CO: *η*^2^*p* = 0.11), whereas neither the treatment effect nor the sex-by-treatment interaction reached statistical significance. These findings indicate that the observed differences were primarily related to sex, with no detectable effect of treatment or sex-dependent treatment response.

As a volumetric surrogate of RV-arterial coupling, we computed the RV SV-to-ESV ratio (SV/ESV). SV/ESV was reduced (Table 2) in metformin-treated animals of both sexes relative to the untreated controls, yielding a significant treatment main effect, with no sex effect or interaction. This framing situates the metformin-treated cohorts within an established coupling-threshold context, in which values approaching 1 indicate progressive uncoupling. Ctrl + Met and PAH + Met in both females and males were the only groups reaching values close to 1. However, no individual pairwise contrasts survived the Tukey correction, indicating a distributed treatment-associated reduction rather than a single group difference.

### 3.6 The flow dynamics of the MPA reveal sex-specific effects of metformin in PAH

All parameters represented in Figure 5 presented significant sex differences (Table 3). Flow parameters even showed sex × treatment differences, suggesting that mean and peak flow are mediated by the influence of the treatment. In addition, peak velocity and MPA area showed treatment (*η*^2^*p* = 0.39 and *η*^2^*p* = 0.62) differences that were assessed by *post-hoc* analysis. In PAH + Met animals of both sexes, peak velocity was lower than that in the corresponding Ctrl + Met group (female: *d* = 2; male: *d* = 1.8). In contrast, the peak and mean flow showed that the Ctrl and PAH groups exhibited similar values, despite the fact that PAH + Met presented higher values than these two groups (Figures 5B,C). Surprisingly, Ctrl + Met showed the highest values for all flow and velocity parameters, indicating that metformin affected the haemodynamic of MPA by enhancing its performance.

**Figure 5:**
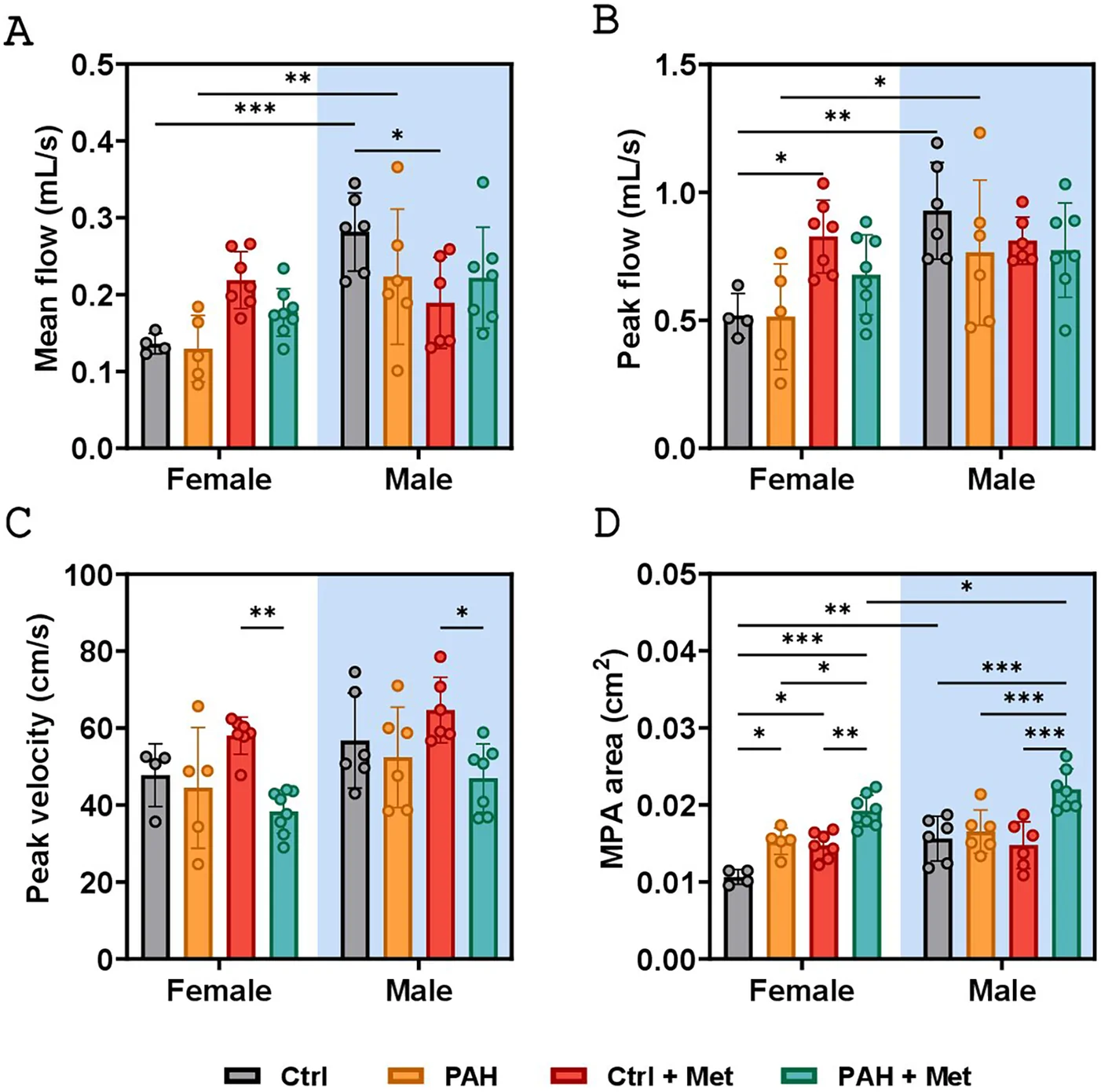
Metformin-treated groups present a similar profile in both sexes. Mean pulmonary artery (MPA): **(A)** mean forward flow per cardiac cycle (mL/s), **(B)** peak instantaneous flow (mL/s), **(C)** peak forward velocity in the ROI (cm/s), **(D)** median MPA cross-sectional area across the cardiac cycle (cm^2^). n: Female: Ctrl = 4, PAH = 5, Ctrl + Met = 7, PAH + Met = 8; Male: Ctrl = 6, PAH = 6, Ctrl + Met = 6, PAH + Met = 7. Data are presented as the mean ± SD. Statistical significance: *p < 0.05, **p < 0.01, ***p < 0.001, assessed using two-way ANOVA *post-hoc* Tukey test.

**Table 3:** Two-way ANOVA of pulmonary-artery phase-contrast flow parameters.

	Two-way ANOVA					
	Sex		Treatment		Sex × treatment	
*n*	*p*-value	*η* ^2^ *p*	*p*-value	*η* ^2^ *p*	*p*-value	*η* ^2^ *p*
Mean flow (mL/s)	<0.001***	0.243	0.372 ns	0.072	0.014*	0.286
Peak flow (mL/s)	0.003**	0.197	0.117 ns	0.133	0.042*	0.179
Peak velocity (cm/s)	0.007**	0.163	<0.001***	0.390	0.991 ns	0.003
MPA area (cm^2^)	0.003**	0.195	<0.001***	0.626	0.102 ns	0.139
Flow-derived CO (mL/min)	0.022*	0.118	0.442 ns	0.061	0.015*	0.219

MPA: main pulmonary artery; CO: cardiac output (flow-derived). n: Female: Ctrl = 4, PAH = 5, Ctrl + Met = 7, PAH + Met = 8; Male: Ctrl = 6, PAH = 6, Ctrl + Met = 6, PAH + Met = 7. Statistical significance: *p < 0.05, **p < 0.01, ***p < 0.001, ns not significant; assessed using two-way ANOVA *post-hoc* Tukey test. One animal excluded after curve-quality review.

The dissociation between velocity and flow is accounted for by a concurrent expansion of the proximal MPA: median MPA cross-sectional area increased in PAH + Met of both sexes (female: *d* = −3.5; male: *d* = −2.62) (Figure 5D). Although both experimental PAH groups presented the same increase, the number of male mice was higher than that of female mice (*d* = −0.52). Because of the volumetric flow rate, the rise in cross-sectional area offsets the fall in peak velocity, leaving peak flow relatively preserved, while the underlying RV stroke output declines. Across animals, the MPA cross-sectional area was inversely associated with peak velocity, supporting the interpretation that reduced peak velocity partly reflects proximal arterial dilation rather than reduced flow alone. PAH + Met from both sexes showed similar profiles for all MPA haemodynamic parameters.

To complete the cardiac MRI characterisation, a validation of SV and CO was carried out (Supplementary Figure S6). A forward SV (Supplementary Table S4) was calculated from the MPA flow measurements to compare against the RV SV obtained through the MRI cine. The same was performed with CO on an animal-by-animal basis (Supplementary Figure S6). Both parameters demonstrated similarity with the MRI cine derived measurements, confirming the accuracy of this technique and validating the results obtained.

### 3.7 Sex-dependent cardiovascular remodelling in lung arterial vessels

To assess pulmonary vascular remodelling, the MWA percentage was stratified by ED into three size categories (Supplementary Figure S7). In large arteries (ED > 200 µm), two-way ANOVA revealed a significant treatment effect (*η*^2^*p* = 0.4) with no significant sex effect or interaction, indicating a sex-independent influence of metformin on medial wall thickening in the largest vessel category (Table 4). *Post-hoc* analysis identified a single significant comparison: male PAH + Met animals showed a higher MWA% than Ctrl + Met animals (*d* = −2.17) (Supplementary Figure S7B) in the large arteries. Numerically, PAH and PAH + Met females show similar MWA% in this size category (*d* = 0.21), consistent with the absence of significant differences observed in the Fulton index and RVSP between these groups.

**Table 4:** Two-way ANOVA of *ex vivo* parameters.

	Two-way ANOVA					
	Sex		Treatment		Sex × treatment	
Parameter	*p*-value	*η* ^2^ *p*	*p*-value	*η* ^2^ *p*	*p*-value	*η* ^2^ *p*
LV Cardiomyocyte area (µm^2^)	<0.001***	0.502	0.043*	0.327	0.025*	0.367
LV Cardiomyocyte number	0.004**	0.345	0.756 ns	0.173	0.253 ns	0.181
RV Cardiomyocyte area (µm^2^)	0.002**	0.381	0.247 ns	0.183	0.166 ns	0.22
RV Cardiomyocyte number	0.04*	0.194	0.274 ns	0.173	0.253 ns	0.181
MWA% >200 µm	0.202 ns	0.06	0.004**	0.383	0.4 ns	0.104
MWA% 100–200 µm	0.361 ns	0.03	0.64 ns	0.052	0.951 ns	0.011
MWA 50–100 µm	0.078 ns	0.096	0.108 ns	0.175	0.15 ns	0.155
RV *Glut*4	<0.001***	0.713	0.016*	0.297	<0.001***	0.522
RV *Hk*2	0.757 ns	0.003	0.717 ns	0.04	0.02*	0.268
RV *Ldh-*1	<0.001***	0.482	0.014*	0.304	0.004**	0.358
RV *Pkm*	<0.001***	0.555	0.191 ns	0.144	0.005**	0.342

Left ventricle (LV) and right ventricle (RV) cardiomyocyte anrea and number by Wheat Germ Agglutinin (WGA) immunofluorescence. n: Female: Ctrl = 3, Ctrl + Met = 4, PAH = 3, PAH + Met = 4; Male: Ctrl = 3, Ctrl + Met = 4, PAH = 3, PAH + Met = 4. MWA%: Medial wall area percentage in elastic stain. n: Female: Ctrl = 4, Ctrl + Met = 4, PAH = 4, PAH + Met = 4; Male: Ctrl = 5, Ctrl + Met = 7, PAH = 4, PAH + Met = 7. Relative fold change in mRNA from RV obtained through RT-qPCR of *slc*2*a*4 (*Glut*4) *hk*2, *pkm* and *ldh-*1. n: Female: Ctrl = 5, PAH = 5, Ctrl + Met = 4, PAH + Met = 4; Male: Ctrl = 5, PAH = 4, Ctrl + Met = 4**, PAH** **+** **Met = 4.** Statistical significance: *p < 0.05, **p < 0.01, ***p < 0.001, ns not significant; assessed using two-way ANOVA *post-hoc* Tukey test.

In the medium arteries (Supplementary Figure S7C), no significant effects were detected for sex, treatment, or their interaction. MWA% was broadly homogeneous across all groups, and in both sexes, PAH + Met was higher (females: 49%–54%; males: 49%–56%) than their Ctrl. In small arteries, female Ctrl + Met animals showed a numerical trend towards lower MWA% compared to PAH females (*d* = −2.03). It can be seen that the PAH females have suffered a thickening of the pulmonary arterial wall, which is less pronounce on the PAH + Met group. On males all groups presented higher values than the control, even the Ctrl + Met which showed significant differences with their female homologous (*d* = −1.65). In the medium arteries (Supplementary Figure S7C), no significant effects of sex, treatment, or their interaction were detected. MWA% was broadly homogeneous across all groups. In both sexes, the PAH + Met groups showed slightly higher MWA% values (females: 49%–54%; males: 49%–56%) than their respective controls. Despite the lack of statistical significance in these histological results at this early stage of PAH, the beginning of the thickening of the pulmonary arteries can be appreciated.

In the small arteries, female Ctrl + Met animals showed considerably lower MWA% than PAH females (*d* = −2.03), consistent with reduced medial wall thickening. Despite the large effect size, this difference did not achieve statistical significance, likely reflecting the limited sample size at this stage of the study. Visual inspection likewise suggested pulmonary arterial wall thickening in PAH females that appeared less pronounced in the PAH + Met group. In males, all groups showed higher MWA% values than the control group, including the Ctrl + Met group, which differed markedly from its female counterpart (*d* = −1.65).

Overall, the data suggest that pulmonary arterial remodelling is beginning to emerge. Together with MPA dilation, reduced MPA peak velocity, lower flow-derived forward stroke volume, and increased RV volumes observed in the same animals, these findings further support the absence of a vascular protective effect of metformin in male PAH animals in this model. This finding, alongside MPA dilation, reduced MPA peak velocity, lower flow-derived forward SV, and elevated RV volumes observed in the same group further supports the absence of a vascular-protective effect of metformin in male PAH animals in this model.

## 4 Discussion

The present study used a factorial 2 × 2 design (Sex × Treatment) in the SuHx mouse model of PAH to characterise the ventricular, haemodynamic and transcriptional response to disease and to metformin in adult young animals. The SuHx protocol reliably induced RV mass gain and elevated pulmonary pressure: the Fulton index was markedly increased in PAH animals of both sexes compared to Control and RVSP was elevated in the corresponding PAH groups (Figures 1D,E). These findings confirm robust model induction and are consistent with the established haemodynamic consequences of SuHx-induced pulmonary vascular remodelling, which produces progressive obliterative arteriopathy and sustained elevation of pulmonary vascular resistance, particularly well characterised in rats (27, 33, 34).

A sex-dependent pattern was observed in body weight (Figure 1). Male PAH animals showed the most pronounced weight loss, particularly at Week 2, whilst the PAH + Met group were more similar to the Ctrl group than to the PAH group, suggesting a weight-preservation effect of metformin in males that was not so evident in females. This was confirmed by the three-way Sex × Treatment × Time, which interaction reached significance in the mixed-effects analysis (*p* < 0.001). Also, food consumption in the PAH + Met group was numerically higher than in PAH in both sexes (Supplementary Figure S1D), suggesting that at least part of the weight-preservation effect of metformin in this model reflects partial recovery of feeding behaviour under drug exposure, a pattern that is opposite to the classical anorectic effect of metformin reported in metabolic-syndrome models (36, 37) and that will require dedicated investigation.

However, once the effect of metformin was examined directly, contrary to the consistent beneficial effects reported in rat models of PAH (20, 21), the primary structural and haemodynamic features of this model were not attenuated in either sex: PAH + Met females showed a numerical but non-significant reduction of the Fulton index relative to PAH females, and PAH + Met males paradoxically showed the highest Fulton index of the cohort. RVSP was likewise unchanged by metformin co-administration in either sex. In mice, metformin failed to reduce RV hypertrophy in either sex, or in males, and was associated with a higher Fulton index than untreated PAH animals. This absence of a beneficial effect on the primary PAH phenotype is consistent with the findings of Goncharov and colleagues (25), who found that metformin at 100 mg/kg/day failed to reduce RVSP, RV hypertrophy, or pulmonary vascular remodelling in male C57BL/6J mice with SuHx-induced PAH, and did not detect benefits of 300 mg/kg administered preventively in an equivalent rat model. Our data extend this negative finding to a mixed-sex cohort and to a higher effective daily dose (410 mg/kg/day in male and 439 mg/kg/day in female Ctrl + Met animals), and are in contrast with the beneficial vascular effect of metformin reported by Dean et al. in female Wistar Kyoto rats with SuHx-induced PAH, in which aromatase inhibition was proposed as a key mechanism (22). Read together, these three studies suggest that the response to metformin in experimental PAH is heterogeneous across species, strains, and sexes, and that the SuHx mouse model may be intrinsically less responsive to metformin than the SuHx rat.

A cellular substrate for the observed RV mass gain was sought by measuring cardiomyocyte cross-sectional area using WGA staining (Supplementary Figure S2). In males, RV cardiomyocyte area tended to be larger in the SuHx groups than in the corresponding controls, consistent with a hypertrophic contribution to the increase in Fulton index, although none of the between-treatment WGA contrasts within a sex reached statistical significance, most likely reflecting the modest per-group histological sample size (*n* = 3–4). In females, RV cardiomyocyte area was essentially unchanged in PAH vs. Control despite a marked increase in Fulton index, pointing to a dissociation between RV mass gain and cardiomyocyte cross-sectional area in this sex. We therefore consider the WGA analysis supportive but not confirmatory of cardiomyocyte hypertrophy as the sole driver of the observed RV mass gain, and note that additional contributions from hyperplasia, extracellular matrix expansion or interstitial fluid, which the present analysis cannot resolve, and deserves dedicated investigation in future studies.

A more robust cellular observation emerged from the between-sex comparisons within metformin-treated groups. In the PAH + Met condition, male cardiomyocytes were 55% larger than female cardiomyocytes in the RV and 51% larger in the LV (Supplementary Figure S7), with comparable sex differences in the Ctrl + Met LV and at the tissue-density level of the PAH + Met RV. These sex differences were absent in the corresponding non-treated groups, where cardiomyocyte area was comparable between males and females in both ventricles, indicating that metformin treatment amplifies or reveals a sex-dependent cardiomyocyte phenotype that is not detectable at baseline. This observation extends the sex-specific cardiomyocyte size phenotypes under metformin suggesting the sex-specific pattern previously reported by McNair et al. (18) in the SuHx mouse model to include both sexes examined in a common experimental framework, and adds cellular-level detail to the broader sex- and substrate-dependent heterogeneity of the metformin response in pulmonary hypertension (21, 22, 24, 38–40). We interpret these cellular observations cautiously, as they arise from limited histological samples and were not the primary endpoint of the study.

The transcriptional profile of glucose handling in the RV showed patterns that were both sex- and treatment-dependent. In male animals, RV *Glut*4 was significantly upregulated in all groups relative to Ctrl. By contrast, in female animals RV *Glut*4 showed a non-significant downward trend across all treated groups. This opposite-direction pattern between sexes for the insulin-responsive glucose transporter is consistent with a differential sensitivity of male and female myocardium to metformin-driven remodelling of substrate-uptake machinery, and mirrors sex differences in cardiac *Glut*4 regulation reported previously (41–43). In the LV, no glycolytic transcript reached significance in any pairwise comparison, indicating that the transcriptional glycolytic response was concentrated in the RV. Recent evidence from the Pulmonary Hypertension through Pulmonary Vascular Disease Phenomics (PVDOMICS) cohort has identified androgenic steroid pathways as sex-differentially associated with RV systolic function in pulmonary hypertension (12), providing clinical support for the biological plausibility of the sex-specific responses documented in our preclinical cohort.

To characterise the second major cardiac energy substrate, we extended the qPCR panel to four genes involved in mitochondrial fatty acid uptake and *β*-oxidation: *Cpt*1*a*, *Ucp*3, *Acadvl* and *Acads*. In the RV, fatty acid oxidation transcripts remained largely unchanged across all groups and both sexes. In contrast, the LV showed a robust and largely uniform downregulation of these transcripts under SuHx and metformin exposure, particularly *Ucp*3 which was reduced in all groups relative to control in both sexes (Supplementary Figures S11B). Together, the glycolytic and fatty acid oxidation transcriptional data describe a coordinated substrate signature in which glucose-handling transcripts are perturbed principally in the RV while fatty acid oxidation transcripts are perturbed principally in the LV (Figure 2 and Supplementary Figure S11). We emphasise that these observations describe the transcriptional level of substrate-uptake and processing enzymes and do not measure enzymatic activity, metabolic flux or cellular energy balance directly. Whether the observed transcriptional pattern reflects a Warburg-like shift toward basal glycolysis at the flux level, as reported in the pressure-overloaded RV of previous experimental PAH studies (6, 8), cannot be established from these data alone and will require direct measurement of glycolytic flux and of fatty acid oxidation in future work.

The concordance between the PAH and metformin transcriptional signatures of glucose and fatty acid substrate-handling genes, rather than an opposition of the two, suggests that metformin, at least at the transcriptional level, engages the substrate-handling machinery in a direction that does not counteract the SuHx-induced reprogramming. This observation illustrates the value of transcriptional screening of substrate-handling genes as a first-line filter for candidate therapies whose downstream metabolic effects can be subsequently validated by flux and enzyme-activity measurements.

Consistent with a substantial glucose-handling component of the RV response, [^18^F]-FDG PET showed elevated myocardial glucose uptake in PAH animals. In the RV, PAH females showed higher uptake than males. Meanwhile in the LV, the PAH + Met males exhibited the highest values, whereas female LV showed a strong but non-significant trend toward elevation in the SuHx groups (Figures 2A–C). Since animals were imaged after a fasting period long enough to suppress insulin and thereby minimise the *Glut*4 contribution to myocardial [^18^F]-FDG uptake (44, 45), the [^18^F]-FDG signal in our study reflects predominantly basal, *Glut*1-mediated glucose transport and its subsequent hexokinase-catalysed trapping as [^18^F]-FDG-6-phosphate, rather than downstream glycolytic flux. This distinction is important because [^18^F]-FDG uptake and glycolytic flux can be dissociated: increased transport and phosphorylation or preserved uptake in the face of reduced downstream flux, are both compatible with the observed PET signal. The preserved-*Glut*1/directionally dimorphic-*Glut*4 transcriptional pattern in the RV, combined with the LV downregulation of fatty acid oxidation transcripts, is compatible with (although not proof of) transcriptional reprogramming toward aerobic glycolysis. Direct assessment of glycolytic flux and of fatty acid oxidation, along with *Glut*1 abundance, will be required to establish the flux consequences of these observations.

An unexpected and clinically relevant finding emerged from the cardiac MRI analysis in our study was the pattern of RV volumetric changes (Figure 3), which did not follow the expected PAH-driven trajectory (13). Whereas untreated PAH animals of both sexes did not show significantly elevated RV EDVi or ESVi compared to Ctrl, the metformin-treated groups did, particularly in females. In female animals, RV ESVi and EDVi was higher in the PAH + Met group while attending to the EF% the highest values were found on the Ctrl + Met (Figure 4). Moving forward in the MRI analysis, these changes continued in the MRI data relating to MPA. Contrary to what has been reported in healthy humans, where males exhibit higher pulmonary artery peak velocity and distinct pulmonary hemodynamic profiles (Figure 4) (49), all female groups in our study presented an enlarged MPA cross-sectional area (Table 2), together with higher peak velocity in the PAH+Met group in both sexes. These findings are methodologically relevant because a reduction in MPA peak velocity in the PAH + Met group could otherwise be interpreted as a favourable reduction in pulmonary vascular resistance; the concurrent MPA dilation and stable forward SV argue instead for proximal pulmonary-arterial remodelling with unchanged effective forward output, which is supported by the combined cine and phase-contrast MRI dataset. The convergence of increased RV ESVi, reduced RV EF%, MPA dilation, and descriptively reduced RV–MPA (SV/ESV) coupling in Ctrl + Met females, none of which was observed in Ctrl + Met males, represents an unexpected sex-specific signal that metformin administration in otherwise healthy animals is associated with volumetric and vascular remodelling of the right heart in females. Although the individual pairwise contrasts are modest in significance and no direct measurement of pulmonary vascular resistance was performed, the pattern is consistent enough across independent MRI-derived readouts to warrant careful consideration and dedicated investigation in future studies.

In rodent SuHx models, cardiac MRI has previously shown progressive RV dilation with preserved SV and EF% during the chronic phase (46, 47), mirroring the RV–pulmonary arterial adaptation described in human PH (40); the concurrent dilation of the proximal MPA observed here extends this picture to the proximal vascular compartment. Consistent with these MPA observations, MWA% analysis stratified by vessel calibre confirmed that metformin did not attenuate the SuHx-induced increase in MWA% in any compartment in either sex, extending the negative pulmonary vascular result from the proximal (MPA) to the distal (arteriolar) compartment (Supplementary Figure S7).

Taken together, these findings do not support a beneficial effect of metformin on the primary structural or haemodynamic features of SuHx-induced pulmonary arterial hypertension in adult young mice of either sex. What the data do support is a set of sex- and ventricle-specific transcriptional, cellular and volumetric responses to metformin: (i) a directionally dimorphic transcriptional response of RV glucose-handling genes to metformin, with male RV upregulation and female RV downward trend of *Glut*4 (Figure 2D); (ii) a coordinated downregulation of LV fatty acid oxidation transcripts under both SuHx and metformin (Supplementary Figure S11); (iii) sex-specific cardiomyocyte size phenotypes revealed under metformin exposure that are not detectable at baseline (Supplementary Figure S2); and (iv) a female-specific pattern of increased RV volumes, reduced RV EF% and MPA dilation in Ctrl + Met animals that emerges as an unexpected safety signal (Table 2). The dissociation between the modest weight-preservation effect (predominantly in males) and the sex-specific cardiac and vascular signals (more pronounced in females) underscores the importance of evaluating metformin effects in both sexes and across multiple organ systems and cautions against the extrapolation of beneficial metformin findings from rat models of PAH to the mouse model, and, by extension, to human PAH, without sex-stratified verification. Whether the sex-specific effects observed here are mediated by sex hormone signalling, AMPK isoform expression differences, or sex-specific differences in metformin pharmacokinetics and tissue distribution (48) remains an open question that will require dedicated mechanistic studies.

This study has several limitations that should be considered when interpreting the results. First, the extensive factorial design imposed practical constraints on per-group sample sizes for individual techniques (particularly histology and qPCR, with *n* = 3–4/group), which limits the ability to detect modest treatment effects and requires that the cellular-level findings be regarded as hypothesis-generating rather than mechanistic conclusions. Second, the transcriptional profile of substrate-handling genes reported here does not measure protein abundance, enzymatic activity, metabolic flux or cellular energy balance directly; direct assessment of glucose and fatty acid flux (e.g., by ^13^C-substrate tracing, lactate and acyl-carnitine quantification, extracellular flux analysis, or myocardial ATP/AMP measurement) will be required to characterise the integrated metabolic function of the myocardium in this model. Direct assessment of substrate flux, as recently shown by ^13^C tracing in PH pulmonary endothelial cells (49) would be required to characterise the integrated metabolic response to metformin in this model. Third, invasive pulmonary vascular resistance measurement and load-independent RV contractility indices were not evaluated, which limits the mechanistic interpretation of the MRI and MPA-flow findings. Fourth, the study was preventive rather than curative in design and did not include a long-term survival follow-up, so the effect of metformin on survival cannot be assessed from these data. Fifth, our histological characterisation of the pulmonary vasculature was based on medial wall area quantification stratified by vessel calibre; a full pathological characterisation of the distal arteriolar remodelling would additionally require enumeration of muscularised vs. non-muscularised arterioles by *α*-smooth muscle actin co-staining, quantification of intimal thickening, and identification of neointimal or plexiform-like lesions, analyses that were beyond the scope of the present study and remain priority directions for follow-up work. Sixth and finally, although the SuHx model recapitulates several features of human PAH, it does not fully capture the genetic, immunological and haemodynamic heterogeneity of the human disease, and the strain- and species-specific responses to metformin discussed above suggest that translational extrapolation should be made with caution.

## 5 Conclusions and prospects

In this study using the SuHx mouse model of pulmonary arterial hypertension, metformin did not attenuate the primary structural (Fulton index) or haemodynamic (RVSP) features of disease in either sex. These findings extend previous negative reports in SuHx rat and mouse models to a mixed-sex cohort and a higher effective daily dose.

Despite the absence of improvement in the primary PAH endpoints, metformin elicited marked sex- and ventricle-specific biological responses. In males, metformin mitigated disease-associated weight loss and was associated with transcriptional changes consistent with enhanced myocardial glucose utilisation, including increased RV expression of *Slc*2*a*4, which encodes the insulin-responsive glucose transporter *Glut*4. In contrast, females exhibited opposite-direction transcriptional responses in the RV. Increased RV volumes, reduced RV ejection fraction and pulmonary artery dilation were also observed in Ctrl + Met females, a finding that warrants further investigation. Additional sex-specific effects were evident at the cellular level, where metformin exposure revealed or amplified differences in cardiomyocyte size that were not apparent in untreated animals. Collectively, these findings demonstrate that metformin exerts substantial biological activity in this model, but that these responses do not translate into improvement of the core structural and haemodynamic manifestations of PAH.

These observations indicate that responses to metformin are strongly dependent on sex and cardiac chamber and that beneficial findings reported in one experimental system should not be assumed to generalise across models without direct verification. They further support the routine inclusion of both sexes and the integration of complementary readouts, including imaging, haemodynamic, transcriptional profiling and histology, in the evaluation of candidate cardio-metabolic therapies for pulmonary hypertension.

Future work will be required to determine whether the sex-specific effects observed here are mediated by sex hormone signalling, AMPK isoform expression differences, or differences in metformin pharmacokinetics and tissue distribution. In parallel, direct assessment of glycolytic and fatty acid oxidation flux alongside transcriptional and imaging analyses would provide a more complete understanding of integrated myocardial metabolism in this model.

## Data Availability

The original contributions presented in the study are included in the article/Supplementary Material, further inquiries can be directed to the corresponding author/s.
