# Supplementary material for: Sex-dependent transcriptional and imaging responses to metformin in young-adult mice with SuHx-induced pulmonary arterial hypertension
Source: Front Cardiovasc Med. 2026 Oct 9;13:1947678. doi: 10.3389/fcvm.2026.1947678 (PMC13690730; doi:10.3389/fcvm.2026.1947678)
Supplement: Supplementary file 1 [file Datasheet1.pdf]

ARRIVE GUIDELINE

Essential 10		Reporting Item	Section
Study design	#1a	Give details of the groups being compared, including control groups. If no control group has been used, the rationale should be stated.<br><br>This study was designed to determine the therapeutical effect of metformin on pulmonary arterial hypertension as a pretreatment. To that aim, we compared four groups of mice: normoxia (Ctrl, normoxia with metformin in the drinking water (Ctrl+Met), Su5416/hypoxia for three weeks (PAH) and Su5416/hypoxia for three weeks with metformin in the drinking water until sacrifice (PAH+Met). These four groups were carried out in both female and male mice, making a total of eight groups.	Methods, Figures, Figure legends.
Study design	#1b	Give details of the experimental unit (e.g., a single animal, litter, or cage of animals).<br><br>The experimental unit was defined as a single animal from each experimental group; it is expressed as "n". From each animal, we performed individual imaging (PET and MRI), hemodynamic measurements and collection of organs for *ex vivo* procedures.<br><br>Despite that all mice in the same cage presented the same amount of water and chow, the food and water consumption was divided by the number of animals in each cage and represented as the average individual consumption of each mouse. Nevertheless, the number of cages is considered the unit for the liquid and food consumption.	Methods, Supplemental material, Figure legends
Sample size	#2a	Specify the exact number of experimental units allocated to each group, and the total number in each experiment. Also indicate the total number of animals used.<br><br>Sample size for each experimental group varied depending on the assay. In accordance with the principle of reduction in animal experimentation, individual animals were used	Methods, Figures, Figure legends, Supplemental, Tables

		for multiple types of analysis (functional, molecular and/or histological) when no cumulative or interfering effects were expected. The total number of animals used for each specific assay is reported in the corresponding figure and it is clearly explained in the supplemental material.	
**Sample size**	**#2b**	Explain how the sample size was decided. Provide details of any a priori sample size calculation, if done.<br><br>No *a priori* sample size calculation was performed. Instead, sample sizes were selected based on prior experience with similar experimental models, practical and logistical constraints, and ethical considerations aimed at minimizing animal use while still enabling the detection of biologically meaningful effects. Additionally, the chosen sample sizes are consistent with those reported in previous studies that successfully identified histological, molecular, and functional cardiac differences in comparable genetic models	Methods, Figures, Figure legends
**Inclusion and exclusion criteria**	**#3a**	Describe any criteria used for including or excluding animals (or experimental units) during the experiment, and data points during the analysis. Specify if these criteria were established a priori. If no criteria were set, state this explicitly.<br><br>Animal exclusion was subjected to the animal death, two female mice (Ctrl+Met and Ctrl+PAH) died during the PET/CT acquisition, making impossible to perform hemodynamic measurements or histological analysis. Also, the hemodynamical surgery that measures the RV pressure entails a risk of mortality which decreases the measure capacity on the animals with PAH.<br><br>The time required for cardiac MRI to produce images, combined with the fact that radioactive scans cannot be carried out on the same day, means that fewer animals undergo MRI than	Methods, Figures, Figure legends

		PET. During the MRI scan it is performed the cardiac MRI and the MPA hemodynamic analysis. In some of the mice it was only performed MPA hemodynamic analysis due to time constraints. Also, some images were discarded from the MRI analysis because of low quality of the cardiac images and MPA flow.<br><br>Despite that PET/CT is a quicker technique, it requires manual intravenously injection of the radiolabelled compound in the tail. On those cases where the compound was not correctly administered, it was excluded from the imaging analysis. Also, as the radioactivity decays, in some cases it was not possible to administer all the animals from each group. These exceptions are subject to the course of the experiment, and it is not possible to predict them due to time constraints and human error.<br><br>The histological evaluation of the disease requires the collection of lungs and heart, which are also valuable tissues for biochemical analysis. To follow the principles of reduction, fewer mice from each group were destinated to histology rather than to biochemical analysis, as histology is a more secure and stablish technique in this laboratory. Except for male lungs from Ctrl+Met and PAH+Met groups, which all lungs were sent to histology. At the time these tissues were collected, the priority was the histological analysis, this changed after the implementation of molecular techniques in our laboratory.<br><br>At RT-qPCR, it was not possible to extract high-quality RNA from all tissues due to the small size of the RV, meanwhile in the LV it was more successful.	
**Inclusion and exclusion criteria**	**#3b**	For each experimental group, report any animals, experimental units, or data points not included in the analysis and explain why. If there were no exclusions, state so.<br><br>The water consumption for the week 2 of PAH female had to be dismissed because the bottle	Methods, figure legends

		was replaced without the necessary records being made.<br><br>At the PET assay, one PAH male and two females from Ctrl+Met and PAH+Met, were excluded from the study because most of the radioactivity was retained in the mice's tails.<br><br>One PAH female was discarded from the MRI cardiac analysis because of low quality of the cardiac images. Also, on the MPA-phase contrast flow two PAH+Met, one female and one male, were discarded as the imaging presented artefacts.<br><br>At the RT-qPCR analysis it was performed a ROUT test, where one female from PAH+Met was excluded from the *Glut4* analysis. Also, analysing *Ldh1* one male PAH sample was excluded following the same criteria.	
**Inclusion and exclusion criteria**	**#3c**	For each analysis, report the exact value of n in each experimental group.<br><br>The exact sample size is reported for each assay in the corresponding Figure or Figure legend, and a groups summary table is placed on the Supplemental materials.	Figure, Figure legends, Supplemental materials
**Randomisation**	**#4a**	State whether randomisation was used to allocate experimental units to control and treatment groups. If done, provide the method used to generate the randomisation sequence.<br><br>No randomisation was carried out during the *in vivo* phases of the study due to the careful monitoring of treatment administration.	Methods
**Randomisatio n**	**#4b**	Describe the strategy used to minimise potential confounders such as the order of treatments and measurements, or animal/cage location. If confounders were not controlled, state this explicitly.	Methods

		To minimize confounding variables, mice from all groups were age-matched and housed under similar conditions.<br><br>Animals that underwent the same drinkable treatment were kept in the same cage. All the bottles containing any treatment were properly marked with visible labels to ensure clarity.	
**Blinding**	#5	Describe who was aware of the group allocation at the different stages of the experiment (during the allocation, the conduct of the experiment, the outcome assessment, and the data analysis).<br><br>Group allocation was known to the researchers during animal selection and experimental procedures, as the cages presented the treatment labels in the bottles. In addition, the permanence in hypoxia conditions makes obvious the induction of the disease. However, outcome assessment and data analysis by different specialists were performed blinded to group identity whenever feasible. This approach helped minimize bias during interpretation of results.	Methods
**Outcome measures**	#6a	Clearly define all outcome measures assessed (e.g., cell death, molecular markers, or behavioural changes).<br><br>Outcome measures were designed to assess cardiac function and structure, metabolic alterations and tissue modifications. These included: (1) Histological analysis of the cardiac and lung tissues by Elastic stain and immunofluorescence trough WGA. (2) Functional parameters such as the right ventricle (RV) systolic blood pressure and the volumes ejected by the heart. (3) Structural characterization of the RV and left ventricle (LV) mass and organ extraction. (4) The metabolic profiling was performed through PET/CT scans and completed by RT-qPCR of the RV and LV. (5) Characterization of the heart performance through MRI.	Methods, Supplemental material, Results

**Outcome measures**	#6b	For hypothesis-testing studies, specify the primary outcome measure, i.e., the outcome measure that was used to determine the sample size.<br><br>No single primary outcome was predefined for sample size calculation. Instead, outcomes measures were chosen based on their biological relevance to the cardiac phenotype investigated.	Methods
**Statistical methods**	#7a	Provide details of the statistical methods used for each analysis, including software used.<br><br>Detailed statistical methods applied are mentioned in the Methods and each Figure legend.	Methods, Supplemental material, Figure legend
**Statistical methods**	#7b	Describe any methods used to assess whether the data met the assumptions of the statistical approach, and what was done if the assumptions were not met.<br><br>It was performed a multiple comparison trough GraphPad Prism (11.0.0) following normal distribution Two-way ANOVA followed by a post-hoc Turkey test. All analyses were independently reproduced in Python (v3.12; pandas, scipy, statsmodels, pingouin) and confirmed to match GraphPad outputs.<br><br>All the specific statistical analysis are described in material and methods.	Material and methods, Figure legend
**Experimental animals**	#8a	Provide species-appropriate details of the animals used, including species, strain and substrain, sex, age or developmental stage, and, if relevant, weight.<br><br>All the mice were *Mus musculus* C57BL/6J from 8-10 weeks old. All the experimental groups were performed on females and males. The details are explained on material and methods section and supplemental material.	Material and methods, Supplemental material, Results, Figure, Figure legend
**Experimental animals**	#8b	Provide further relevant information on the provenance of animals, health/immune status,	Material and methods, Figures

		genetic modification status, genotype, and any previous procedures.<br><br>Animals were purchased from Charles Rivers Laboratories (Charles River Laboratories, Wilmington, MA, USA) and housed in the Animal Facility at Centre for Cooperative Research in Biomaterials (CIC biomaGUNE).	
**Experimental procedures**	#9a	For each experimental group, including controls, describe the procedures in enough detail to allow others to replicate what was done, how it was done, and what was used.<br><br>The detailed experimental procedures are documented and can be found in the Methods and Supplemental material sections.	Material and methods
**Experimental procedures**	#9b	Timing and frequency of procedures<br><br>Intraperitoneal injections of Su5416 were administered once a week, metformin treatment was administered *ad libitum* in the drinking water. All the *in vivo* measurements were performed a single time after the three weeks of disease induction, for one week. There is more information on the methods section.	Material and methods, Figures, Figure legend
**Experimental procedures**	#9c	Where procedures were carried out (including detail of any acclimatisation periods).<br><br>All the procedures were carried out on the Animal Facility or Radiochemistry Facility at Centre for Cooperative Research in Biomaterials (CIC biomaGUNE).	Material and methods
**Experimental procedures**	#9d	Rationale for procedures<br><br>The experimental procedures carried out were approved by the authorized Ethical Committees and followed the standard established protocols.	Material and methods
**Results**	#10a	For each experiment conducted, including independent replications, report summary/descriptive statistics for each experimental group, with a measure of	Material and methods, Figure, Figure legend

		variability where applicable (e.g., mean and SD, or median and range).<br><br>The description and statistic for each experiment conducted in mice is detailed in the Methods and corresponding Figure legend.	
**Results**	**#10b**	If applicable, for each experiment conducted, including independent replications, report the effect size with a confidence interval.<br><br>For all comparisons data is presented as mean±SD as detailed in Figure legends and methods, except for the body weight ratio and Phase-aligned cine MRI volume curves where the data is represented as mean±SEM.<br><br>Effect sizes for post-hoc comparisons were calculated in Python from the pooled error term of the corresponding two-way ANOVA and are reported as Cohen's $d = (M_1 - M_2) / \sqrt{MS\_residual}$, as it is described in materials and methods.	Material and methods, Results
