# Supplementary material for: Sex-dependent transcriptional and imaging responses to metformin in young-adult mice with SuHx-induced pulmonary arterial hypertension
Source: Front Cardiovasc Med. 2026 Oct 9;13:1947678. doi: 10.3389/fcvm.2026.1947678 (PMC13690730; doi:10.3389/fcvm.2026.1947678)
Supplement: Supplementary file 2 [file Supplementaryfile1.docx]

Supplementary Material

# 1 Supplementary materials and methods

## 1.1 Cardiac magnetic resonance imaging

As indicated in materials and methods, per-mouse LV and RV volume curves comprise 15 time points spanning one cardiac cycle. Because retrospective gating yields cardiac cycles of variable length, the position of end-diastole relative to the first acquired frame differs across animals. To enable averaging and statistical comparison of curves across the cohort, each ventricle's curve was phase-aligned independently by circularly shifting it so that its maximum value (end-diastole) sat at the first phase point. Aligned curves were then resampled to 100 equally spaced phase points using piecewise cubic Hermite interpolating polynomial (PCHIP) interpolation, with the curve treated as cyclic (the last point is contiguous with the first). PCHIP was chosen over cubic spline interpolation because it preserves monotonicity between adjacent samples and does not generate spurious extrema, which is critical when the maximum and minimum of the resampled curve are used as scalars in downstream analysis.

The scalars (EDV, ESV, SV, EF) were confirmed to reproduce the conventional frame-pick scalar values (i.e., max and min of the 15-frame curve) to within numerical precision. All subsequent group-level analyses were performed on these curve-derived scalars; analyses are described in the Statistical analysis subsection below. In a closed circulation at steady state, LV and RV SV must agree by mass conservation. As an a priori segmentation quality control, the LV-derived and RV-derived SV of each animal were compared. Animals were flagged for re-tracing if |LV SV − RV SV| / mean (LV SV, RV SV) exceeded 25 %, a threshold beyond which physiological mechanisms cannot plausibly account for the discrepancy.

For this analysis, 48 mice were studied divided into the four experimental groups. Only one animal was excluded from the cardiac MRI analysis after curve-quality review (**Figure S4**). Note systematic RV < LV stroke-volume offset (manually segmented under-tracing of the RV), and Panels B/C show functional parameters determined by curve-vs-endpoint, showing agreement with bias ≈ 0. Three additional animals had isolated outlying values and were excluded only from the affected parameter, at the level of ventricular volumes.

To characterise ventricular dynamics in addition to chamber geometry, the first time-derivative of each phase-aligned volume curve was computed by central finite differences. From the derivative we extracted peak ejection rate (PER), defined as the magnitude of the most negative dV/dt; peak filling rate (PFR), the most positive dV/dt; and the corresponding time-to-peak values as the phase positions at which these extrema occurred. PER and PFR are reported both in absolute units (µL per percent of cardiac cycle) and after normalisation to end-diastolic volume (EDV per percent of cycle), the latter to distinguish intrinsic kinetic effects from geometric chamber-size effects.

# 2 Supplementary Figures and Tables

Figure S1. Weekly monitoring of mice. Body weight trajectories over 4 weeks in (A) female and (B) male mice measured individually; (C) Bottle weight and (D) food consumption were measured per bottle-cage, so the values are mean for the number of animals in each case. Body weight data are shown as mean ± SEM, while food consumption and bottle weight are shown as mean ± SD. n: Female: Ctrl = 12, Ctrl+Met = 9, PAH = 12, PAH+Met = 9; Male: Ctrl= 12, Ctrl+Met=8, PAH=12, PAH+Met=8. Data are presented as mean ± SD. Statistical significance: *** p < 0.001, using two-way ANOVA post-hoc Tukey’s test.

Figure S2. Wheat Germ Agglutinin immunofluorescence (A) Representative images of Wheat Germ Agglutinin (WGA) staining in RV tissues. WGA (green) and DAPI (blue). Scale bar, 50 μm; Quantification of cardiomyocyte area (µm^2^) in the (B) left ventricle (LV) and the (C) right ventricle (RV); Number of cardiomyocytes in the (D) LV and the (E) RV. n: Female: Ctrl = 3, Ctrl+Met = 4, PAH = 3, PAH+Met = 4; Male: Ctrl = 3, Ctrl+Met = 4, PAH = 3, PAH+Met = 4. Data are presented as mean ± SD. Statistical significance: * p < 0.05, ** p < 0.01, *** p < 0.001, using two-way ANOVA post-hoc Tukey's test.

**Figure S3.** Relative fold change in mRNA from the left ventricle (LV) obtained through RT-qPCR of (A) *slc2a4* (*Glut4*), (B) *hk2*, (C) *ldh-1*, and (D) *pkm*. Values are presented as mean ± SD. Statistical significance: * p < 0.05, ** p < 0.01, using two-way ANOVA post-hoc Tukey’s test.

Figure S4. Sex contrasts within each treatment group. Mean difference (Female − Male) with 95% confidence interval for the six primary cardiac MRI parameters: End diastolic volume (EDV) (µL), end systolic volume (ESV) (µL), ejection fraction (EF) (%). Negative values indicate that females have smaller volumes (or lower EF) than males. Point estimates and 95% cardiac index (CI) (mL(min/m^2^)) are derived from Tukey HSD on the Sex × Treatment two-way ANOVA (k = 2, df = 39). Sample sizes per cell: Female 5–9, Male 5–6. Bold mean-difference labels indicate Tukey-adjusted p < 0.05.

Figure S5. Cardiac MRI parameters across all experimental groups. From the right ventricle (RV) (A, C, E) and the left ventricle (LV) (B, D, F) there are presented: (A and B) End systolic volume index (ESVi), (C and D) End diastolic volume index (EDVi), (E and F) Cardiac output index (COi). n: Female: Ctrl=4, Ctrl+Met=7, PAH=4, PAH+Met=9; Male: Ctrl = 5, Ctrl+Met = 5, PAH = 5, PAH+Met = 6. Values are presented as mean ± SD. Statistical significance: * p < 0.05, ** p < 0.01, using two-way ANOVA post-hoc Tukey’s test.

Figure S6. Pulmonary-artery phase-contrast magnetic resonance imaging. (A) Pulmonary artery forward stroke volume (SV). (B) Comparison between the cardiac output (CO) obtained through the cardiac MRI and volume calculations and the CO obtained through calculations over the PA flow (striped columns). n: Female: Ctrl = 4, Ctrl+Met = 7, PAH = 5, PAH+Met = 9; Male: Ctrl=6, Ctrl+Met = 6, PAH = 5, PAH+Met = 6. Statistical significance: * p < 0.05, ** p < 0.01, using two-way ANOVA post-hoc Tukey’s test.

Figure S7. Medial wall area percentage (MWA%) in elastic stain. A) Representative images of the largest external diameter (ED) by elastic stain, scale bar 50 µm. Pulmonary artery medial wall area percentage stratified by vessel ED in (B) Large arteries with ED > 200µm, (C) Medium arteries with 100–200µm ED, (D) Small arteries with 50–100µm. Data are presented as the mean ± SD. n: Female: Ctrl = 4, Ctrl+Met = 4, PAH = 4, PAH+Met = 4; Male: Ctrl = 5, Ctrl+Met = 7, PAH = 4, PAH+Met = 7. Statistical significance: ** p < 0.01, using two-way ANOVA post-hoc Tukey’s test.

**Figure S8. Validation of curve-derived cardiac MRI quantification against endpoint measurements.** **(A)** Left ventricle (LV) and right ventricle (RV) stroke volume (SV, µL) per-animal. The solid line is the line of identity, and the dashed lines indicate ±10%. (B) Bland–Altman comparison of end diastolic volume (EDV) and end systolic volume (ESV) on the LV and RV. The x-axis shows the mean of the two methods and the y-axis their difference (curve – endpoint data analysis, µL); the solid line is the mean bias (+4.0 µL) and the dashed lines the 95% limits of agreement (+23.0 to −15.0 µL). (C) Bland–Altman comparison for LV and RV ejection fraction (EF), with the difference expressed in percentage points (curve– endpoint); mean bias −2.3 pp and 95% limits of agreement +4.6 to −9.3 pp. One animal (Male PAH+Met) was excluded after curve-quality review.

**Figure S9. Per-animal pulmonary-artery flow waveforms.** (A, C, E, G) Female (B, D, F, F) male Signed through-plane flow (mL/s) in the main pulmonary artery across one cardiac cycle, phase-aligned to systolic onset (frame 0), shown for every animal (thin coloured lines) with the group mean superimposed (bold black line). n: Female: Ctrl = 4, PAH = 5, Ctrl+Met = 7, PAH+Met = 8; Male: Ctrl = 6, PAH = 6, Ctrl+Met = 6, PAH+Met = 7. Dashed grey lines denote studies excluded from quantitative analysis on image-quality grounds.

**Figure S10. Group-averaged pulmonary-artery flow waveforms.** Mean signed through-plane flow (mL/s) across the phase-aligned cardiac cycle (frame 0 = systolic onset), shown separately for (A) females and (B) males, group sizes are indicated in the panel legends. Curves illustrate the systolic ejection waveform and its modulation by disease and metformin; quantitative indices derived from these data (peak velocity, mean velocity, peak and mean flow) are reported in **Table 1** and **Table 2**. n = 49. Values are presented as SEM ± SD.

**Figure S11.** Relative fold change in mRNA from right ventricle (RV) (A, C, E and D) and (LV) (B, D, F and H) obtained through RT-qPCR of (A, B) *cpt1a*, (C, D) *ucp3*, (E,F) *acads*, and (G, H) *acadvl*. Values are presented as mean ± SD. Statistical significance: * p < 0.05, ** p < 0.01, *** p < 0.001, using two-way ANOVA post-hoc Tukey’s test.

Table S1: Number of animals contributing to each experimental procedure, by sex and treatment group

| **Procedure** | **Female** | | | | **Male** | | | | **Total** |
| --- | --- | --- | --- | --- | --- | --- | --- | --- | --- |
|  | **Ctrl** | **PAH** | **Ctrl+Met** | **PAH+Met** | **Ctrl** | **PAH** | **Ctrl+Met** | **PAH+Met** |  |
| Cardiac MRI (cine volumes, EF, indexed volumes, RV–PA coupling) † | 4 | 4 | 7 | 9 | 5 | 5 | 5 | 6 | 45 |
| PA phase-contrast flow (velocity, area, flow-derived SV/CO) | 4 | 5 | 7 | 8 | 6 | 6 | 6 | 7 | 48 |
| [^18^F]-FDG-PET (myocardial SUV) | 5 | 5 | 7 | 7 | 4 | 4 | 5 | 5 | 42 |
| RV systolic pressure (closed-chest catheter) | 4 | 5 | 5 | 4 | 6 | 5 | 4 | 4 | 37 |
| Fulton index (RV/[LV+S]) | 6 | 6 | 4 | 5 | 6 | 5 | 4 | 4 | 40 |
| Pulmonary vascular morphometry (medial wall area) | 4 | 4 | 4 | 4 | 5 | 4 | 8 | 8 | 35 |
| Cardiomyocyte histology (WGA: area, number) | 3 | 3 | 4 | 4 | 3 | 3 | 4 | 4 | 28 |
| RV qPCR | 5 | 5 | 4 | 5 | 5 | 6 | 4 | 4 | 38 |
| LV qPCR | 6 | 6 | 4 | 4 | 6 | 6 | 5 | 5 | 42 |

*A total of 82 mice (42 female, 40 male) were studied. Group sizes vary by procedure because no single animal underwent every measurement; values are the number of animals analysed per group (after exclusion of pre-identified outliers, flagged in the source data). Study design: in-vivo endpoints (cardiac MRI, [18F]-FDG, PA phase-contrast flow) were acquired in shared animals, frequently over consecutive days; terminal Ctrl and PAH endpoints (RV systolic pressure, Fulton index, pulmonary vascular morphometry, RV qPCR) were obtained from separate animal cohorts; metformin-treated animals contributed to both in-vivo and terminal measurements.*

*† From cardiac MRI volumes and function one mice excluded on image-quality review. Two additional animals excluded per-parameter outlier exclusion.*

*PA flow, two animals from Female Control and Female PAH+Met groups were excluded for off-vessel/blurred phase-contrast images, and a third from Male PAH+Met excluded on image-quality review.*

**Table S2A:** Liquid consumption per mouse (mL/mouse/week)

| **Sex** | **Treatment** | **N (mice)** | **Mean ± SD (mL/mouse/week)** |
| --- | --- | --- | --- |
| **Female** | **Ctrl** | 6 | **19.8 ± 8.1** |
| **Female** | **Ctrl+Met** | 9 | **27.3 ± 1.3** |
| **Female** | **PAH** | 6 | **20.0 ± 1.4** |
| **Female** | **PAH+Met** | 9 | **25.9 ± 7.9** |
| **Male** | **Ctrl** | 6 | **22.4 ± 2.8** |
| **Male** | **Ctrl+Met** | 8 | **31.6 ± 2.3** |
| **Male** | **PAH** | 6 | **38.2 ± 8.6** |
| **Male** | **PAH+Met** | 8 | **21.6 ± 1.7** |

*Mean ± SD calculated from n=3-9 animal per cage. One drinking measurement was excluded from the analysis (female control group, week 2) due to a confirmed recording error in the laboratory notebook. Ctrl, PAH: n=3-6 mice/cage, 1 cages/group; Ctrl+Met, PAH+Met: n=8-9 mice/cage, 2 cages/group.*

**Table S2B:** Food consumption per mouse (g/mouse/week)

| **Sex** | **Treatment** | **N (mice)** | **Mean ± SD (g/mouse/week)** |
| --- | --- | --- | --- |
| **Female** | **Ctrl** | 6 | **18.6 ± 0.9** |
| **Female** | **Ctrl+Met** | 9 | **20.5 ± 1.7** |
| **Female** | **PAH** | 6 | **16.9 ± 1.5** |
| **Female** | **PAH+Met** | 6 | **21.6 ± 3.4** |
| **Male** | **Ctrl** | 6 | **23.4 ± 1.0** |
| **Male** | **Ctrl+Met** | 8 | **26.2 ± 0.9** |
| **Male** | **PAH** | 6 | **20.9 ± 6.2** |
| **Male** | **PAH+Met** | 8 | **22.1 ± 2.5** |

*Ctrl, PAH: n=5-6 mice/cage, 1 cages/group; Ctrl+Met, PAH+Met: n=3-9 mice/cage, 2 cages/group. Mean ± SD across three weeks. The group female Ctrl and PAH only include mean values of a single cage due to a reported laboratory error.*

Table S3. Molecular markers studied by RT-qPCR in mouse right ventricle (RV) and its primer sequence.

| **Gene** | **Target protein** | **Forward sequence** | **Reverse sequence** |
| --- | --- | --- | --- |
| ***rpl13a*** | Ribosomal protein L13a | ctctcaaggttgttcggctg | aggagtccgttggtcttgag |
| ***slc2a4*** | Solute carrier family 2 member 4 (*Glut4*) | ggaacactggtcctagctgt | atcatgccacccacagagaa |
| ***hk2*** | Hexokinase 2 | ctgtttgaccacattgccga | caccacagccacaatgtcaa |
| ***ldha*** | Lactate dehydrogenase 1 | tggcttggaaaatcagtggc | aagagacttcagggagacgc |
| ***pkm1*** | Pyruvate kinase isoform 1 | ctggaatgaatgtggctcgg | taagcgttgtccagggtgat |
| ***Ucp3*** | Uncoupling protein 3 |  |  |
| ***Cpt1a*** | Carnitine palmitoyltransferase 1a | gggctactcagaggatggac | tgccaaaggtgtcaaatggg |
| ***Acads*** | Acyl-Coenzyme A dehydrogenase short chain | gggcaagaaggaagacaagc | ctcggcatacttcacagcac |
| ***Acadvl*** | Acyl-Coenzyme A dehydrogenase long chain | tctgcaaggctgtatggaca | attgatccagagcctgcact |

**Table S4.** Phase-contrast MRI of the main pulmonary artery.

| **Parameter** | **Female** | | | | **Male** | | | | |
| --- | --- | --- | --- | --- | --- | --- | --- | --- | --- |
|  | **Ctrl** | **PAH** | **Ctrl+Met** | **PAH+Met** | **Ctrl** | **PAH** | **Ctrl+Met** | **PAH+Met** |  |
| **n** | **4** | **5** | **7** | **8** | **6** | **6** | **6** | **7** |  |
| **HR (bpm)** | 517 [508–525] | 490 [480–518] | 522 [513–529] | 509 [505–521] | 514 [498–543] | 526 [519–528] | 538 [529–542] | 527 [525–532] |  |
| **MPA area (cm²)** | 0.011 [0.010–0.011] | 0.015 [0.015–0.016] | 0.015 [0.014–0.016] | 0.019 [0.018–0.021] | 0.016 [0.013–0.018] | 0.016 [0.015–0.017] | 0.015 [0.013–0.017] | 0.022 [0.020–0.024] |  |
| **Peak velocity (cm/s)** | 51.4 [46.9–52.3] | 48.8 [34.3–48.9] | 58.7 [58.1–60.7] | 39.6 [34.8–43.1] | 51.9 [50.0–65.3] | 53.2 [40.9–59.7] | 61.9 [58.6–69.2] | 50.8 [38.8–52.5] |  |
| **Peak flow (mL/s)** | 0.50 [0.48–0.54] | 0.53 [0.37–0.65] | 0.86 [0.71–0.91] | 0.68 [0.58–0.81] | 0.90 [0.76–1.06] | 0.75 [0.54–0.87] | 0.78 [0.74–0.86] | 0.75 [0.70–0.88] |  |
| **Mean flow (mL/s)** | 0.14 [0.14–0.14] | 0.15 [0.12–0.16] | 0.21 [0.20–0.25] | 0.18 [0.18–0.19] | 0.29 [0.24–0.32] | 0.23 [0.20–0.30] | 0.23 [0.20–0.26] | 0.25 [0.20–0.25] |  |
| **Mean velocity (cm/s)** | 14.1 [13.7–14.4] | 11.5 [10.4–13.9] | 16.0 [15.0–17.1] | 10.0 [9.6–10.3] | 15.8 [15.3–18.5] | 15.8 [13.0–18.6] | 17.9 [16.5–18.7] | 13.0 [11.9–13.5] |  |
| **Forward SV (µL)** | 15.4 [15.1–16.2] | 19.2 [13.3–19.5] | 24.2 [21.6–27.6] | 20.7 [20.2–21.5] | 34.0 [27.8–34.8] | 27.7 [21.8–34.8] | 25.7 [22.2–29.0] | 23.0 [22.4–27.7] |  |
| **Net SV (µL)** | 14.9 [14.5–15.9] | 14.9 [10.5–19.5] | 24.1 [20.5–27.1] | 20.2 [18.5–21.3] | 33.7 [27.7–34.6] | 26.2 [21.5–29.8] | 20.0 [14.8–26.4] | 20.9 [17.6–25.5] |  |
| **Backward fraction (%)** | 3.1 [1.9–3.8] | 1.6 [0.0–22.4] | 1.2 [0.8–2.3] | 1.4 [0.3–5.4] | 0.6 [0.4–0.7] | 5.7 [0.7–14.3] | 16.8 [2.8–33.1] | 5.5 [3.0–8.5] |  |
| **CO flow (ml/min)** | 7.9 [7.6–8.2] | 7.2 [5.5–9.6] | 12.6 [11.1–13.9] | 10.3 [9.7–11.0] | 17.3 [14.6–18.7] | 12.5 [11.3–15.1] | 11.0 [7.9–14.3] | 13.6 [10.1–14.1] |  |

*Values are group median (n as in the header row). Flow cohort = 49 animals (three studies excluded for image quality). MPA, main pulmonary artery; SV, stroke volume; CO, cardiac output.*

*Forward SV and CO are flow derived. Backward fraction = backward/forward flow × 100. Two-way ANOVA (Sex × Treatment): peak velocity Treatment p = 0.0001; MPA area Treatment p < 0.0001; peak flow Treatment p = 0.117 (NS — velocity–flow dissociation). Tukey HSD post-hoc in main text.*
